# Supplementary material for: Discovering sparse control strategies in neural activity
Source: PLoS Comput Biol. 2022 May 27;18(5):e1010072. doi: 10.1371/journal.pcbi.1010072 (PMC9140285; doi:10.1371/journal.pcbi.1010072)
Supplement: S1 Text — Appendices A-J. (PDF) [file pcbi.1010072.s001.pdf]

S1 Text: Discovering sparse control strategies in neural activity

Edward D. Lee[1*], Xiaowen Chen[2], Bryan C. Daniels[3]

1 Complexity Science Hub Vienna, Vienna, Austria
2 Laboratoire de physique de l'École normale supérieure, CNRS, PSL Université, Sorbonne Université, Université de Paris, Paris, France
3 School of Complex Adaptive Systems, Arizona State University, Tempe, AZ, USA

* edlee@csh.ac.at

A Sketching an overview of stochastic mapping and information geometry

The question of control is closely linked to how neural states map to behavior. When the mapping is not deterministic, this is a problem of stochastic mapping of the behavioral state Z to the neural state Y as pictured in Figure A. A simple example comes from the context of sensory coding, where "grandmother" neurons represent increasingly complex patterns of sensory stimuli as information is funneled up a hierarchy [1, 2]. In this case, the mapping between Y and Z is precise. For example, we take Y to be the firing rate of some neuron and Z to be the similarity of the stimulus to one's grandmother. Since firing rate is a mean of a noisy measurement, it is more appropriate to interpret the average firing rate to characterize a distribution $P(Y)$, e.g. a Poisson distribution for the number of times a neuron fires within a time frame. Varying the mean activity level of a single neuron traces out a curve in the space of possible distributions $P(Y)$ in Figure AC. In the space of possible perceptions $P(Z)$ (Figure AB) given by $P(Z) = \sum_Y P(Z|Y)P(Y)$, we trace out a corresponding curve. Likewise, the manifolds of stochastic neural activity and behavior are connected by the relationship that accounts for imprecision and uncertainty.

Going beyond single neurons, sparse sets of neurons together may span the range of possible sensory inputs. In sparse coding, a basis set of neurons are extracted from measuring neural response to visual stimuli, where neural activation represents abstract image features such as edges, corners, and contours [3, 4]. The idea of sparse coding is that typical visual scenes can be represented with activity in only a few neurons, with each neuron representing a separate high-level feature. This constitutes a multidimensional $P(Y)$ specified by the behavior of each neuron instead of the unidimensional one in the grandmother example. It is the joint activity amongst these distributed components that captures the whole image, an idea that has been extended in various ways in the literature [5, 6].

Variation in sensitivity of neural activity is described by the local dependence of the neural activity distribution $P(Y)$ on parameters specifying its form. As pictured in Figure AA, "stiff" directions correspond to large changes in $P(Y)$ being caused by small changes to neural parameters [7, 8]. In the "sloppy" directions, changes to neural parameters are ineffectual, and only dramatic perturbations cause a comparable change in $P(Y)$. The relative elongation of the local information geometry disappears when encoding places no special importance on any neural subgroup such that each plays a commensurate role, as in some forms of population coding [9, 10]. Since behavior $P(Z)$

depends implicitly on $P(Y)$, the parameters underlying neural activity also define the sensitivity of behavior. In this way, local information geometry informs us about the sloppiness of control through response to perturbation.

More formally, a perturbation to underlying neural activity is reflected in a modified probability distribution $\tilde{p}(s) = p(s) + \Delta(s)$ over discrete states s. A unique measure of distinguishability, the Kullback-Leibler divergence D_{KL} [11, 12], between the original distribution and the modified one reduces to

$$D_{\mathrm{KL}}[p||\tilde{p}] \approx \frac{1}{2}\sum_s \frac{\Delta(s)^2}{p(s)} = \frac{1}{2}\sum_s \sum_{ij} F_{ij} dv_i(s) dv_j(s). \tag{A.1}$$

Then, the complete set of perturbations is given by the Hessian F_{ij}, known as the Fisher information matrix (FIM). It describes the local sensitivity of $p(s)$ to perturbations along vectors dv, which may be constrained by what is accessible in the experiment or model. Because moving from one basis to another is a linear operation, the particular form of the perturbations are not as important as the fact that the set should span the possible set of perturbations. By exploiting this property of analyticity, we can in principle reduce the complexity of the problem by measuring a single set of experimental perturbations.

The eigenvectors of F_{ij} in Eq A.1 capture the second dimension of control from Figure 1. When stiff directions involve changes to only few neurons, collective sensitivity is concentrated. Such a possibility reflects centralized control, which would simplify the multiplicity and complexity of control nodes. Since we expect real world examples to display a range of structure depending on organism and function, this provides a methodology for inferring such variation from data.

As a more intuitive formulation of Eq 5 which is the expansion in Eq A.1 applied to collective synchrony, we could assign to each possible collective configuration a "synchrony energy" $\mathcal{E}$ such that its probability can be written $\phi \propto \exp[-\mathcal{E}]$. Such an effective energy represents a coarse-graining over the microscopic states corresponding to a collective configuration. Then, Eq 5 can also be written as the limiting quantity involving the change in energies $\Delta\mathcal{E}$ under such a perturbation, the variance $\lim_{\epsilon\to 0}\left(\left\langle \Delta\mathcal{E}^2\right\rangle - \left\langle \Delta\mathcal{E}\right\rangle^2\right)/\epsilon^2$ [13]. This is a measure of how differently the log-probability of each collective configuration changes. Thus, the Fisher information is proportional to the variance of the effective energy such that more sensitive directions of change are ones that maximally scatter the collective distribution.

B Numerical solutions to inverse maxent problem

In principle, the maxent formulation presents a unique mapping from statistical correlations to model parameters such that there is no parameter fitting in the usual sense. By definition, the maxent model captures only steady-state statistics and not short-timescale dynamics or fine-scale control mechanisms, and we use it to probe control at the level of such statistics.

The problem of determining the fields $h_{m,k}$ and couplings J_{mt} that closely match mean activity and pairwise correlations is known as the inverse maxent problem [14, 15]. In practice, however, limitations to numerical precision and finite-sample noise mean that fitting the parameters for even moderately sized systems is not without ambiguity. With this ambiguity in mind, we present the different approaches we use to solve the inverse problem for the pairwise maxent and independent models.

The first method, which we focus on in the main text, allows us to identify a sparse interaction network between neurons mirroring the sparse structural connectivity of the neural connectome [16]. The parameters of the maxent model are initialized at zero and

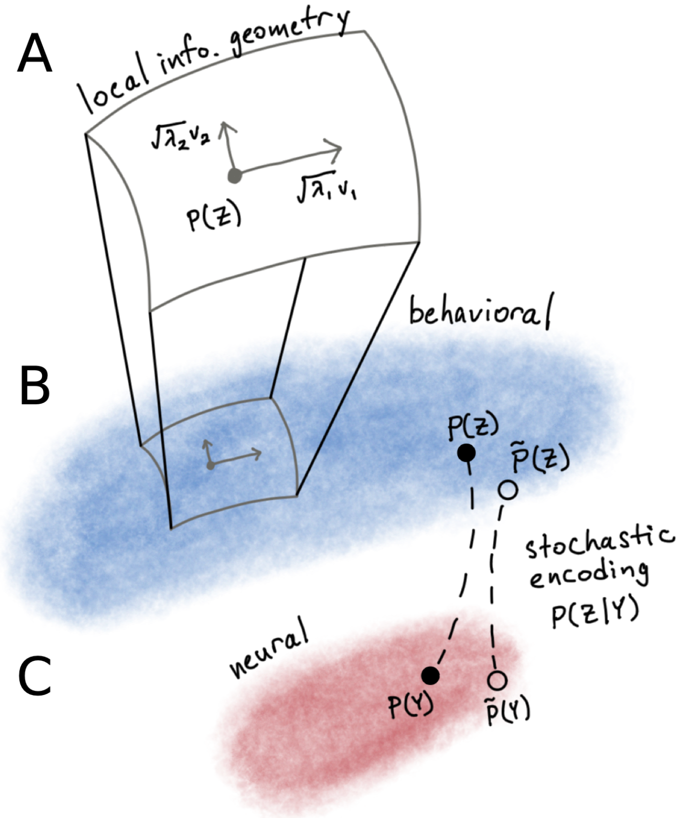

Fig A. (A) Local curvature of manifold of probability distributions $P(Z)$ is captured by the Fisher information matrix (FIM). FIM eigenvectors v_i represent neural perturbation axes. Eigenvalues λ_i denote strength of curvature, or inverse sensitivity. (B) Set of possible behavioral distributions $P(Z)$ as a high-dimensional manifold. (C) Neural behavior represented as ensemble $P(Y)$ over states of activity Y. Relationship between $P(Y)$ and behavioral distribution $P(Z)$ is given by stochastic encoding $P(Z|Y)$ that maps neural activity to behavior. In a given experiment, this mapping may also depend on environmental conditions such as in our study case of *C. elegans* that are bead immobilized.

are updated individually as to maximally increase the likelihood of the data at each step [17, 18]. This learning procedure is stopped once the model reproduces the constrained observables within experimental variability, estimated by bootstrapping random halves of the data. If experimental variability is relatively large, this training procedure can return a sparse interaction matrix J_{mt}, where the average number of edges per vertex is 14 for the entire *C. elegans* neural network. We show the results of such a procedure in Figures 2 and B.

We note that signalling pathways have been found between neurons in the *C. elegans* brain that are not synaptically connected. For reviews, see references [19] and [20], and for a distinct example relevant for short-time behavior see reference [21].

The second method we use for comparison relies on the Monte Carlo Histogram (MCH) approach [18], which is an approximate gradient descent algorithm. At each step, the parameters are simultaneously updated by an amount that is proportional to the difference between the corresponding observable calculated from the model and the data. We obtain a solution that is within a norm error threshold

$$\left(\sum_{k=-1}^{1}\sum_{m=1}^{N}[r_k^{\text{data}}(s_m) - r_k(s_m)]^2 + \sum_{m<t}^{N}(\langle s_m s_t\rangle_{\text{data}} - \langle s_m s_t\rangle)^2\right)^{1/2} < \frac{1}{2}, \tag{B.2}$$

where the cutoff is arbitrarily set to obtain a relatively fast and close fit to the statistics of the data. Unlike the first method, MCH returns a dense network of connections since all couplings are updated at every iteration til convergence. While we find that the resulting model aligns well with the features of the data, including collective synchrony, this procedure does not make realistic assumptions about the topological structure of the underlying physical network. For these solutions, we again find a signal for distinguishing pivotal neurons in the uniformity of columns and rows, but it is not as evident. We plot the column and row uniformities in Figure J.

We also consider an independent neuron model. When solving the corresponding maxent model, we penalize large fields by maximizing the log-likelihood $\log \mathcal{L}_i$ for each spin i along with a penalty such that the total cost function $\mathcal{C}_i$ is defined as

$$\mathcal{C}_i \equiv -\log \mathcal{L}_i + \frac{1}{\sigma}\sum_{k=-1}^{1} h_{i,k}^2 \tag{B.3}$$

because large fields make it especially costly to calculate the FIM accurately. Indeed, a sparse cost function, one where the cost scales with the absolute value of the fields, does not sufficiently penalize large fields, rendering it infeasible for our implementation of the FIM calculation. With the constraint given in Eq B.3, the goal is then to find the fields such that

$$h_{i,k}^* = \min_{h_{i,k}} \mathcal{C}_i. \tag{B.4}$$

To determine the weight σ, we compute the cost function $\mathcal{C}_i$ in Eq B.3 for a range of σ. At small σ, the quadratic penalty dominates and the function approaches a large constant. At large σ, we recover the original maximum likelihood problem. We set $\sigma = \sigma^*$ as is determined by the midpoint between these two extremes for the cost function averaged over all spins i. Typically, this is in the interval $\sigma^* \in [2, 10]$. These steps return an independent model of neurons, where the biases of the most extreme neurons have been tempered.

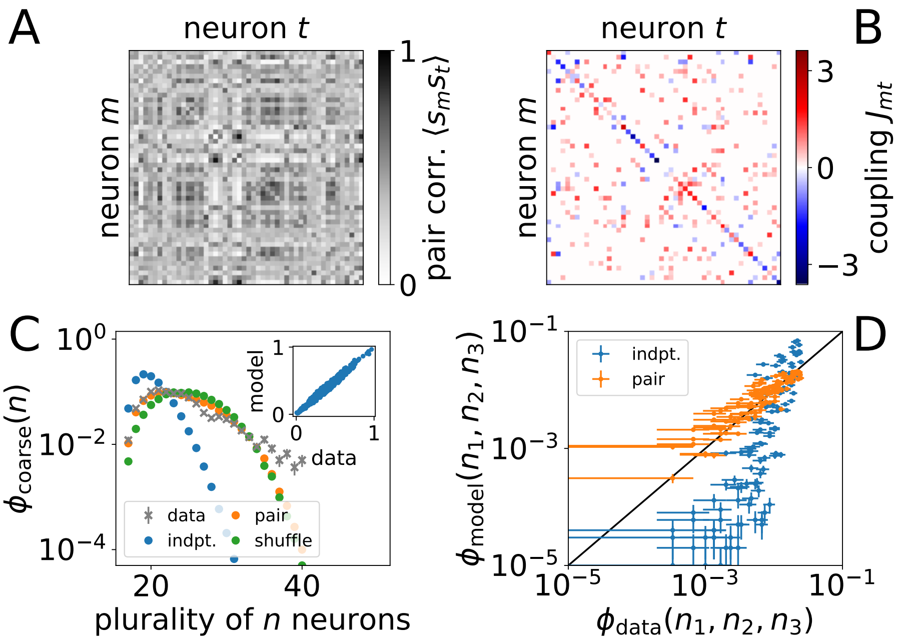

Fig B. Maxent model fit overview for experiment 154139. See Figure 2 for experiment 170419 and panel explanations.

C Neural synchrony & behavior

In the main text, we propose neural synchrony, or how strongly neural states coincide, as a measure of collective neural behavior and of worm behavior. Although synchrony has been used as a theoretical measure of collective neural activity [22], it has not been shown to be related to behavior. To make this connection, we consider a recent data set on neural activity and worm posture (curvature) and locomotion (velocity in forward and backward directions) from reference [23].

As a first step, we discretize calcium level derivatives into three states (up, down, flat) in a way that maximizes the flatness of the distribution that any given neuron is in one of the three states. This procedure ensures that the discretization does not favor any particular state over another although it is not our more precise procedure for extracting discrete states — that requires a good estimate of the derivative, which is not currently possible with mobile worms. We likewise discretize velocity $v(t)$ and curvature $c(t)$ symmetrically about 0, choosing a bin size of $K = 5$ (although our conclusions do not depend on this strongly). Then, we calculate the mutual information (MI) between synchrony and the two measures of behavior separately. For example, for velocity, this would be

$$MI[\phi; v] = \sum_t p(v, \phi) \log_2 \frac{p(v, \phi)}{p(v)p(\phi)}, \tag{C.5}$$

$$= H[v] + H[\phi] - H[v, \phi], \tag{C.6}$$

where H is the entropy. To correct for finite-sample bias, we use the standard NSB estimator to calculate the individual and joint entropies [24]. For comparison, we compute the mutual information between each individual neuron and the measures of behavior as a test of whether or not collective information is greater. These steps define a procedure for determining whether synchrony can be considered a proxy of behavior.

We show in Figure C MI for the two types of collective synchrony compared with

individual neurons. While most experiments show significant MI between synchrony and worm behavior (blue and orange), it is not always the case. However, where the dependence is relatively small or zero within error bars, we also find that MI with individual neurons is small (green and red), suggesting that there are some experiments where neural activity does not say much about behavior. This may be from instrumental noise, which substantially more important for mobile worms. Interestingly, there can be a few highly informative individual neurons that are as informative as fine-grained sychrony, but the typical neuron is already about as informative as coarse-grained sychrony. Thus, synchrony is informative about behavior though coarse-grained synchrony is not any more informative than the typical neuron.

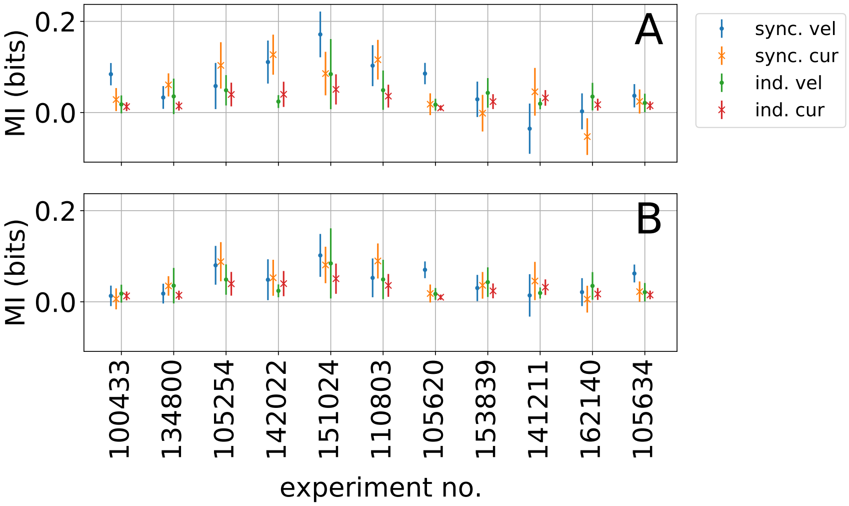

Fig C. Mutual information (MI) between worm behavior (velocity, curvature) and neural activity (synchrony, individual neuron activity) with data from reference [23]. (A) Fine-grained synchrony. (B) Coarse-grained synchrony. Note that individual neuron MI's do not change between panels A and B. Error bars for synchrony MI are given by the NSB estimator applied to estimates of the joint and independent entropies separately [24]. This means the estimator can be negative but is reassuringly zero within error bars in every case except one. Error bars for individual neuron MI are a standard deviation over the neurons.

D Calculation of Fisher information matrix (FIM)

To calculate the entries of the FIM, we rely on a Monte Carlo Markov Chain (MCMC) sample of the distribution of neural states $p(s)$, which we then coarse grain to approximate the distribution over collective synchrony ϕ. With the distribution, we then calculate its Kullback-Leibler divergence under perturbation, which is straightforward to do by altering the statistical correlation functions calculated on the MCMC sample according to Eq 4. Relating this change to a change in the fields $\{h_i\}$ and couplings $\{J_{ij}\}$ is a linear matrix problem in the perturbative limit. With the corresponding change in the parameters, we calculate the change in energy $\Delta E(\{s_i\})$ for each configuration s sampled, the coarse graining of which maps onto the corresponding effective energy of the system. This algorithm is specified in more detail in the supplementary information of reference [13].

Overall, the calculation of the FIM is an expensive computational task where we must obtain each entry of the $(N^2 - N) \times (N^2 - N)$ FIM which are averages over K

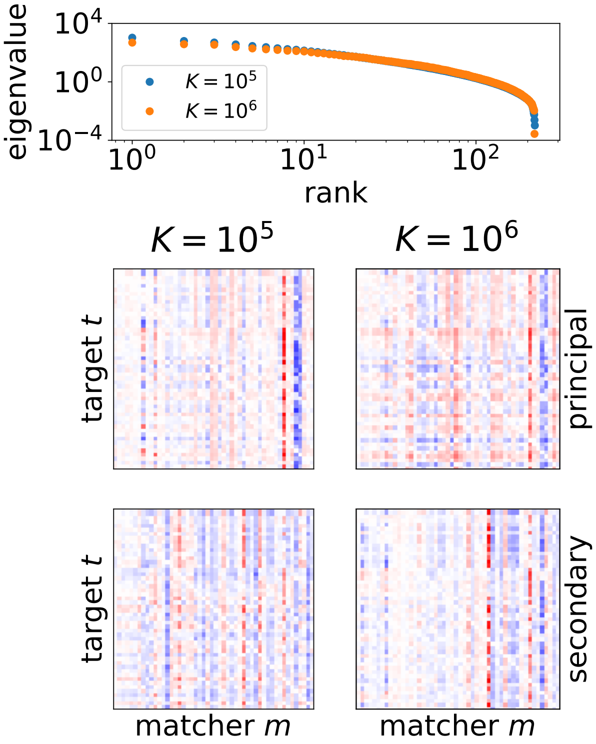

Fig D. Comparison of (top) eigenvalue spectrum and (bottom) eigenmatrices calculated from Monte Carlo samples of sizes $K = 10^5$ and $K = 10^6$. Eigenvalue spectrum for $K = 10^5$ is an average calculated over 10 different Monte Carlo samples. Top two eigenvectors are taken from a single sample as an example, and the two columns share similar, if not identical, features.

MCMC samples for each of $|\phi|$ coarse-grained statistics. To parallelize such a calculation, we combine custom code with the Metropolis algorithm implemented in the ConIII Python package [15]. We relied on a number of computational resources including local workstations and computing clusters at the Santa Fe Institute, University of New Mexico, and Vienna Scientific Computing (VSC).

In the main text, we show results from MCMC samples of size $K = 10^5$ and compare a few cases with a larger samples of size $K = 10^6$ to verify that we can obtain a good approximation of FIM properties with the smaller sample having fixed $\epsilon = 10^{-4}$. At the very least, perturbation magnitude should be smaller than the inverse of the MCMC sample size. We verify that our estimates of the FIM entries converge within a relative tolerance of 0.1% when compared with a larger perturbation of $\epsilon = 2 \times 10^{-4}$. Furthermore, this lower bound on the strength of perturbation is complemented from above by two additional bounds. First, it is unlikely that the system can relax back from a large perturbation. Second, a sufficiently large perturbation breaks the linear assumptions we make. These upper bounds might not separate with the lower bound for measurement, which would pose a problem for measurement, but this is a question that must be answered experimentally.

The example of the eigenvalue spectra in Figures 4 and P involve an average over 10 samples of size $K = 10^5$ for the pairwise maxent model the shuffled null model. See Figure D for a comparison of spectral properties of the FIM between the two sample sizes considered.

E Analyzing eigenmatrices

We measure the strength of vertical striations in the eigenmatrices by comparing the sum of row and column uniformities as defined in Eq 7. In Figures G, H,I, and J, we show uniformity for 3 additional neuron subsamples for the main approach discussed in the text, the independent model, the shuffled null model, and the MCH solutions, respectively. For pairwise perturbations, we find matrices strongly biased to large column sum norms compared to rows. Given the nature of the pairwise perturbations that we consider, that means that perturbations localized to a particular neuron (in contrast with perturbations that would impact each of the neighbors in turn) would have a dominant impact on the collective outcomes. This is a feature of localized control because it means that turning off all local synaptic connections, or turning them up in a way that makes the matcher more similar to all other neurons, is effective.

As a more direct measure of this, we can analyze the subspace of the FIM corresponding to perturbation of each neuron at a time, fixing m and iterating over all t. As we show in Figure E, the principal eigenvalues extracted from single neuron perturbations are correlated with the fraction of MC samples in which the same neuron has unusually strong column uniformity. Thus, two different measures of pivotal neurons, one based on single neuron subspaces and another based on the structure of the entire system, align and show that the strong collective tendencies that we find in the data do not prevent individual neurons from playing important roles.

To determine the scaling of the rank-ordered eigenvalue spectrum, we fit the function detailed in the main text and reproduced here

$$\lambda(z) = C z^{-\alpha} e^{-z/\bar{z}}; \qquad z \leq Z_{\max} \tag{E.7}$$

for eigenvalue λ with rank z by performing least-squares minimization on the logarithmic differences. However, there is no *a priori* guarantee that the spectrum of eigenvalues is full rank and the tail of the spectrum may reflect numerical precision errors. As a heuristic, we apply a hard cutoff when the logarithmic slope falls below -3 and do not fit any points for rank above the cutoff.

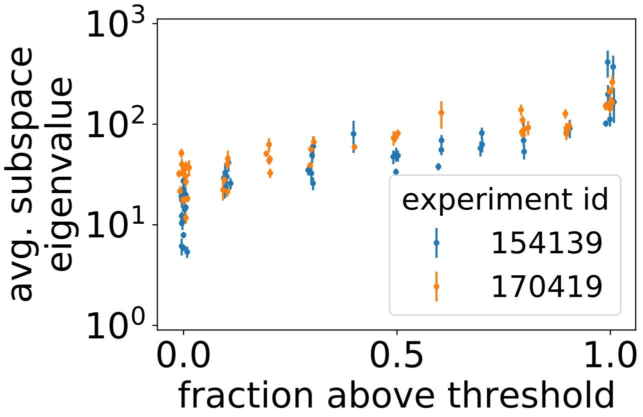

Fig E. Principal subspace eigenvalues vs. fraction above column uniformity threshold of 99-percentile (compare with Figure 6). Subspace corresponds to diagonal blocks of FIM for fixed matcher index m, which is used in Figure 4C to order neurons. Average taken over ten MC samples with standard error of the mean shown. This means that neurons often above threshold tend to have large collective sensitivity, linking the two measures of pivotal strength. Points are randomly offset on x-axis for visibility.

Additionally, numerical precision makes it difficult to estimate the smallest eigenvalues and thus the exact value of the cutoff of the spectrum. We take an effective cutoff $Z_{\max}$ when eigenvalues $\lambda < 10^{-7}$, minuscule in comparison with the typical principle eigenvalue in the range of 10^2 to 10^4. As is visible in our fit in Figure 4, Eq E.7 with a hard cutoff hews very well to the averaged FIM spectrum. In contrast, a simple exponential decay, having set $\alpha = 0$, cannot capture the scaling form that we observe. We show an overview of the truncated power law fit exponents and exponential tails in Figures 5 and F.

We compare our findings for the pairwise maxent model with null models including an independent model for neural activity and one where the couplings are randomly assigned to a pair called "coupling shuffled." While the independent model gives qualitatively different results, we find that shuffling the couplings amongst pairs preserves the qualitative features of the FIM.

F Classes of perturbations

We distinguish between two principal classes of perturbations in the main text denoted "observable" and "natural" or "canonical," taking for the latter the nomenclature used in Amari's textbook on information geometry [12]. Essentially, these distinguish between perturbations defined in the space of correlations compared with perturbations defined in the space of parameters. Since perturbations in either representation can be transformed to one other by a linear operation, there is no *a priori* reason to prefer one basis over another. Theoretical treatments tend to consider the canonical picture because of the underlying intuition that they correspond to physical forces.

In physics experiments, it is possible to access directly quantities such as fields, couplings, and temperature to directly modify the Hamiltonian. With a phenomenological model of the neural network, however, we do not expect that perturbations protocols simply map to canonical perturbations. This means that for the natural perturbations we define in Eq 4, there is a corresponding representation in terms of the fields $\{h_i\}$ and couplings $\{J_{ij}\}$, but it will generally be a complicated combination of many changes across the system (see Figures 3 and K). In this sense, the

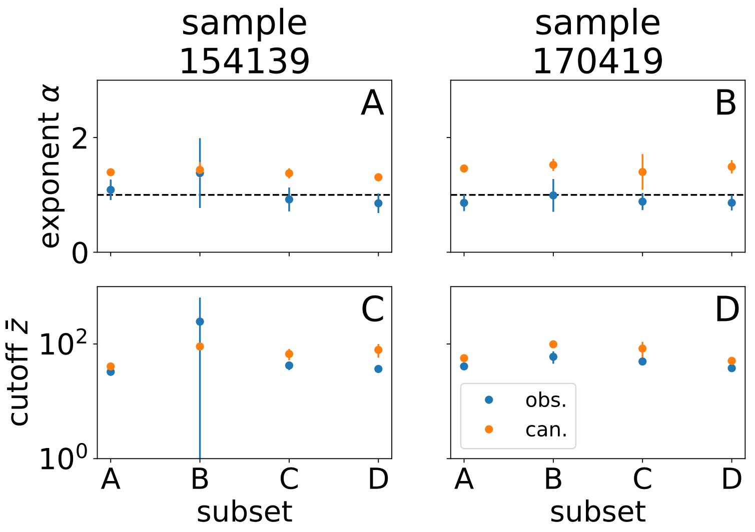

Fig F. (A, B) Power law exponent from fitting FIM eigenvalue spectra comparing observable and canonical perturbations. (C, D) Scale of exponential tail $\bar{z}$.

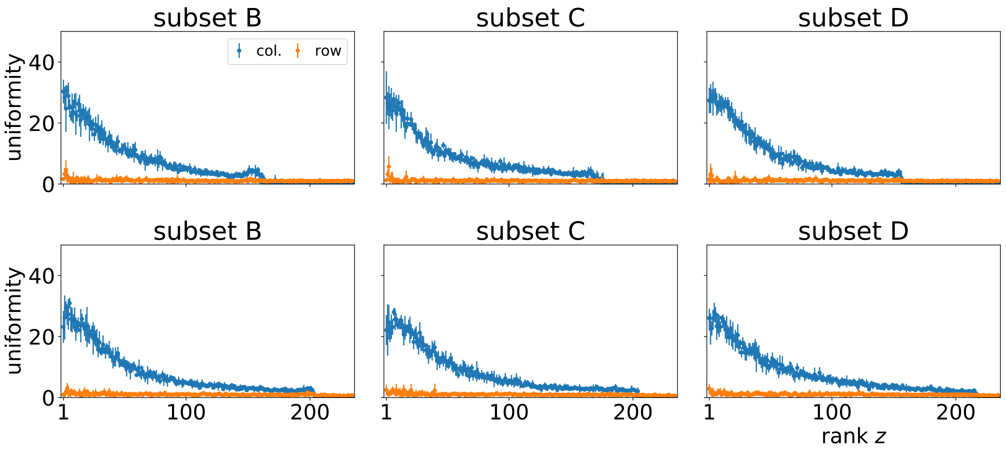

Fig G. Uniformity of eigenmatrices for random neural subsets of $N = 50$ neurons. We show averages over Monte Carlo samples. Error bars show one standard deviation. (top row) Subsets from experiment 154139. (bottom row) Subsets from experiment 170419.

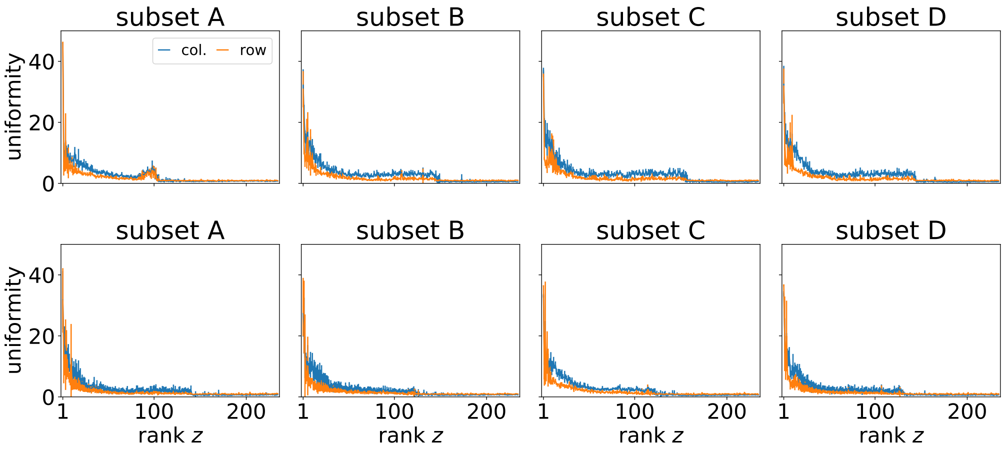

Fig H. Uniformity of the eigenmatrices for independent model. There is no dramatic separation between column and row uniformity. See Figure G for details.

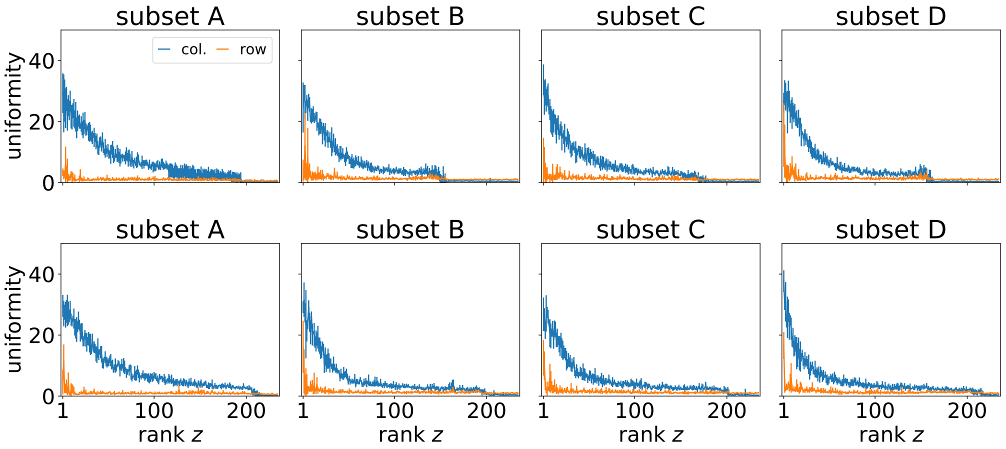

Fig I. Uniformity of eigenmatrices after shuffling couplings. Permutation mostly preserves separated column and row uniformities. See Figure G for details.

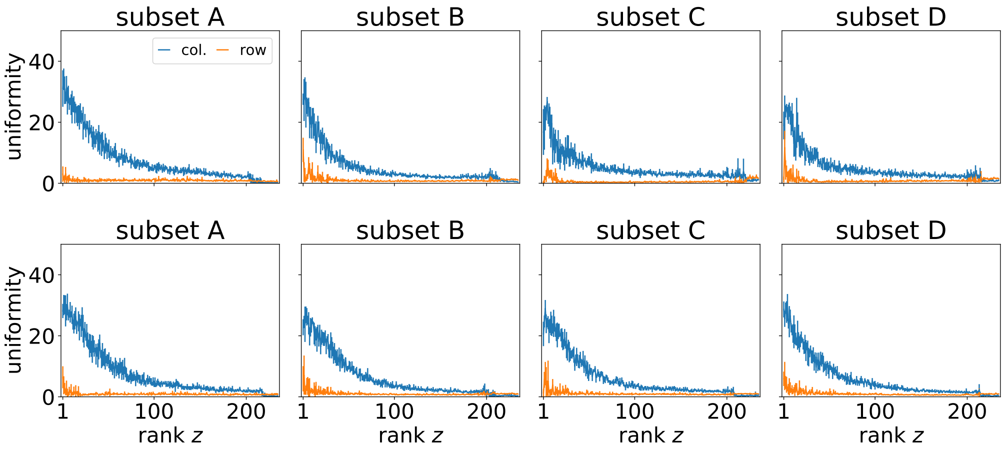

Fig J. Uniformity of eigenmatrices for MCH solutions. See Figure G for details.

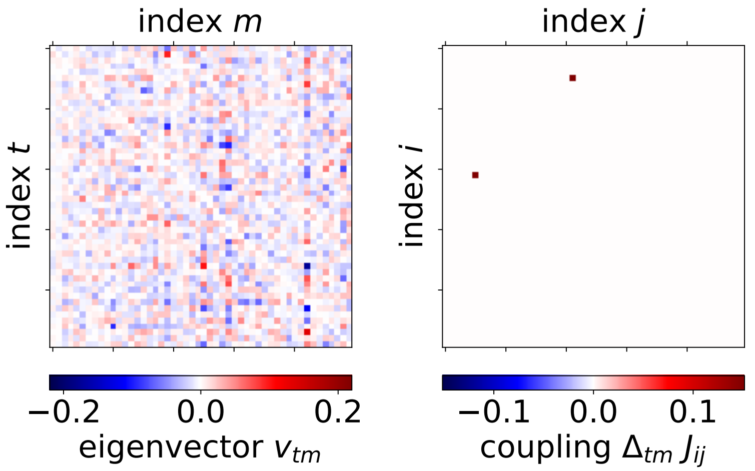

Fig K. Example of simple coupling perturbation mapped to space of replacement rule. Compare with Figure 3C.

pairwise maxent model serves as a representation of our inference process rather than a literal model of the neural network.

Besides the distinction between types of perturbations, we have a choice of which protocols to consider within each space. In the main text, we discuss individual neuron perturbations relative to neighbors as a way of approaching closed-loop control. However, other experimental protocols might be of interest such as probabilistic clamping to a fixed external reference frame that may be increasing, decreasing, or flat. An infinite variety of such variations are possible, and it may be the case that other representations turn out to be more appropriate for capturing simple experimental interventions. Though a complete basis can always, in principle, be transformed from one set of perturbations to another, such transformations may be difficult to perform accurately from limited and potentially noisy data extracted from experiments.

For the sake of completeness, we additionally compute the FIM for the example of clamping each neuron to an external reference frame—akin to clamping to an imaginary neighbor that is always in the up, down, or flat state. For the case of $K > 2$ possible states of the neuron, clamping a neuron to a single state involves specifying the relative chance in the probabilities that the neuron is in the remaining $K - 1$ states. We consider the case where clamping the neuron to a particular state y changes the probabilities of the remaining two configurations in a way that traces out the geodesic towards $p_y \equiv p(s_m = y) = 1$ in the two-dimensional simplex (Figure O). Eventually, experimental results may suggest more empirically grounded ways of accounting for the relative change in probabilities.

Taking the described formulation, the local perturbation for the matcher neuron changes its bias as

$$\begin{aligned}\tilde{p}_x &= p_x - \epsilon(1 - p_y)\frac{\cos(\pi/2 - \theta_y)}{\cos(\theta_y - \pi/6)},\\ \tilde{p}_y &= (1 - \epsilon)p_y + \epsilon,\\ \tilde{p}_z &= p_z - \epsilon(1 - p_y)\frac{\cos(\pi/6 + \theta_y)}{\cos(\theta_y - \pi/6)}.\end{aligned} \tag{F.8}$$

In Figure L, we show a summary of results from such a protocol on the pairwise maxent model. We find that eigenvalues decay faster with rank when considering observable perturbations compared with natural perturbations for both fine and coarse collective

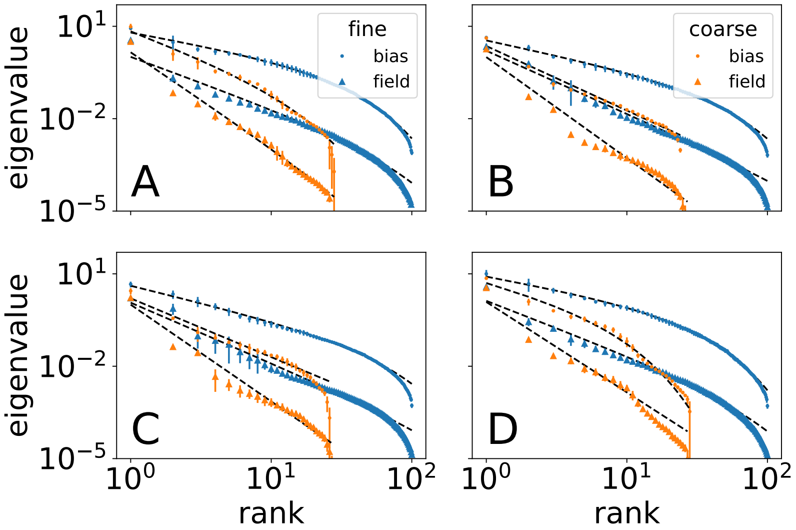

Fig L. FIM eigenvalue spectra from field and bias perturbations for experiment 170419. We compare spectra for ϕ_{fine} with ϕ_{coarse}. Panel letters indicate different neuron subsets of $N = 50$ and averages over Monte Carlo samples with error bars representing standard deviations.

synchrony. Across the maxent models we consider and both measures of synchrony, we consistently find that the decay exponent is faster for natural perturbations, in alignment with our results for pairwise perturbations.

G Relating observable and canonical perturbations

In the main text, we primarily focus on perturbations to the observed correlations, but we also find consistent differences between observable and natural perturbations. As we show in Figure 4, the spectrum tends to decay faster for canonical perturbations and they are smaller in general. To gain some insight into these differences, we consider the two types of perturbations for a simpler case of a binary neuron characterized by a mean activity level.

Consider states s with probabilities $p(s) = \exp[-E(s)]/Z$ coarse-grained into a distribution $\phi(k) = \sum_{|s|=k} p(s)$, where the notation $|s| = k$ means that there are k spins in the majority. Under the replacement rule, the probability of neuron i being in one configuration p_i is modified to become

$$\begin{aligned}\tilde{p}_i &= (1-\epsilon)p_i + \epsilon \\ &= p_i + (1-p)\epsilon.\end{aligned} \tag{G.9}$$

Note that the derivative that returns the appropriate is of the form $-d/d[\log(1-p_i)] = (1-p_i)d/dp_i$. This would correspond to our observable perturbation, whereas the canonical perturbation would be with respect to the Langrangian multipliers, the fields and couplings for the pairwise maxent model.

The quantity of interest is the "score function," or the expectation value of the second derivative of the probability distribution that gives us the entries of the FIM,

$$-\left\langle \frac{\partial^2 \log \phi}{\partial[\log(1-p_i)]^2} \right\rangle. \tag{G.10}$$

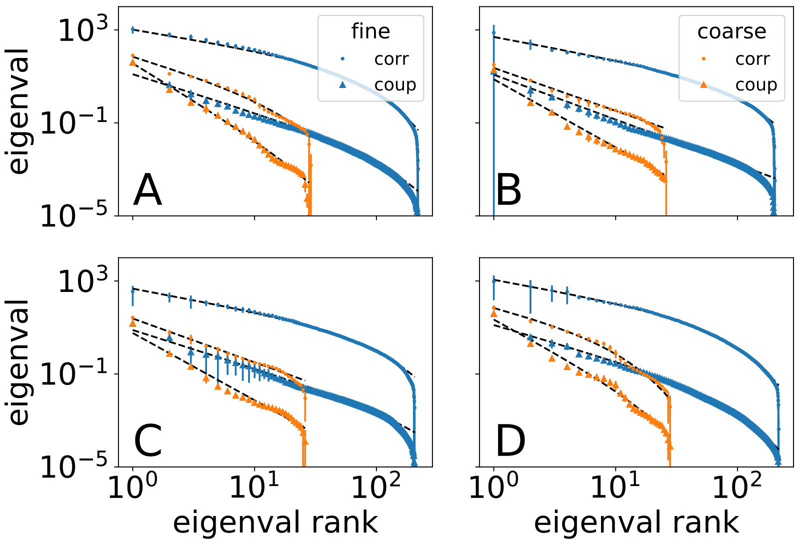

Fig M. Power law with truncated exponential fit as dashed black lines to mean eigenvalue spectra for experiment 170419. Sensitivity for ϕ_{fine} in blue and ϕ_{coarse} in orange. Error bars show standard deviation over 10 Monte Carlo samples used to calculate FIM over 4 different subsets of $N = 50$ neurons shown in each panel.

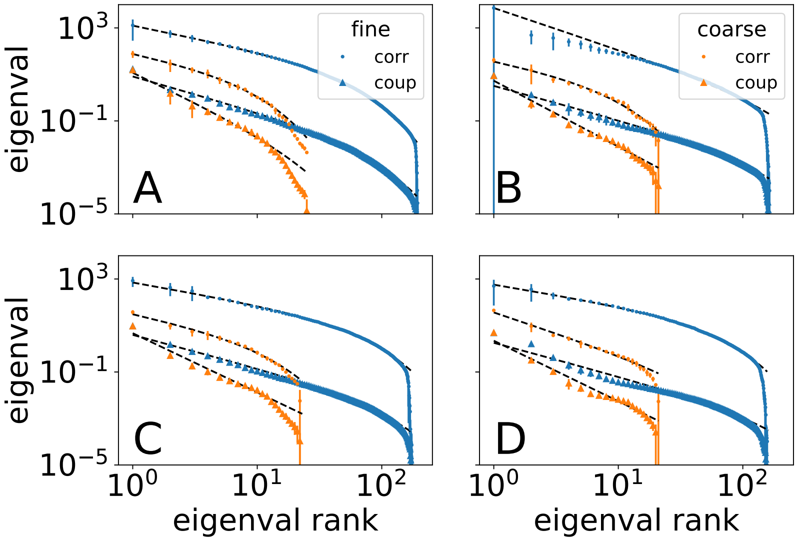

Fig N. Power law with truncated exponential fit to mean eigenvalue spectra for experiment 154139. See Figure M for more details.

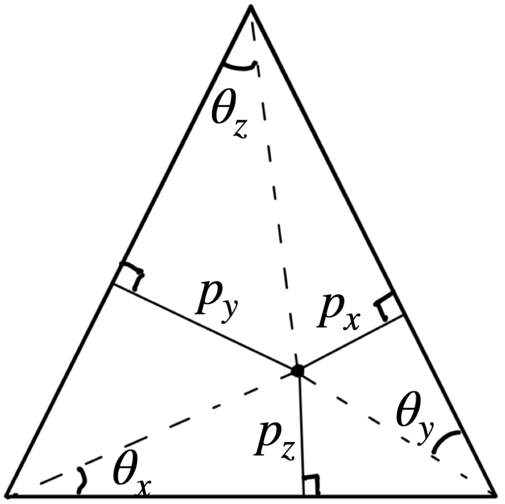

Fig O. Probability simplex for three-state neuron with probabilities of being in each of three states denoted by p_x, p_y, and p_z such that $p_x + p_y + p_z = 1$.

The terms involving $\log\phi$ are of course the susceptibilities of the kth correlation function with the spin i. Since ϕ is a coarse-grained version of $p(s)$, we expect that the derivatives will reduces to linear combinations of correlation functions.

To simplify notation, we take the term inside the brackets having defined $y_i \equiv \log(1-p_i)$ to obtain

$$\begin{aligned}\frac{\partial^2 \log\phi}{\partial y_i^2} &= \frac{\partial}{\partial y_i}\left[\frac{\partial h_i}{\partial y_i}\frac{\partial \log\phi}{\partial h_i}\right] \\ &= \frac{\partial^2 h_i}{\partial y_i^2}\frac{\partial \log\phi}{\partial h_i} + \left[\frac{\partial h_i}{\partial y_i}\right]^2 \frac{\partial^2 \log\phi}{\partial h_i^2},\end{aligned} \tag{G.11}$$

where we have introduced the field h_i for neuron i. By taking the derivative with respect to the field, we have incurred a term proportional to the curvature in the change of variables as well as the square of the jacobian bringing us from y_i to h_i.

Now, we calculate the jacobian terms. Note that we are only considering the domain where the relationship between $\log(1-p_i)$ and h_i is analytic, otherwise we would have to deal with branch cuts. Given this caveat, we differentiate

$$\frac{\partial y_i}{\partial h_i} = \frac{1}{p_i - 1}\frac{\partial p_i}{\partial h_i}. \tag{G.12}$$

Using the fact that $p_i = (\langle s_i\rangle + 1)/2$ and that the derivative is the susceptibility, or the variance of spin i, we obtain

$$= \frac{1}{\langle s_i\rangle - 1}\left(1 - \langle s_i\rangle^2\right) \tag{G.13}$$

$$= -(1 + \langle s_i\rangle). \tag{G.14}$$

Then, the second derivative is

$$\frac{\partial^2 y_i}{\partial h_i^2} = -\left(1 - \langle s_i\rangle^2\right). \tag{G.15}$$

Thus, the maxent model allows us to explicitly calculate the jacobian in terms of linear response quantities that tell us how the observables change under a small perturbation of the fields.

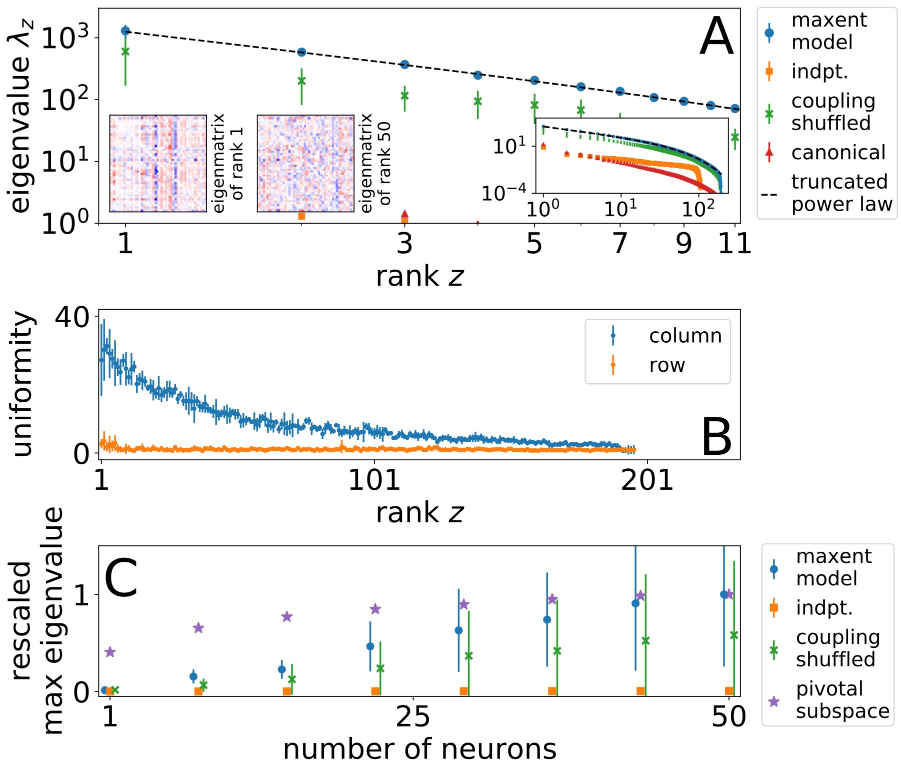

Fig P. FIM overview for worm experiment 154139. In main text, we show corresponding plots for experiment 170419 in Figure 4.

Now, let us go back to the score function in Eq G.10. Note that because all the above quantities are already ensemble averages, we can factor them out of the brackets in Eq G.10. This means that we have

$$\frac{1}{\langle s_i \rangle + 1} \left[\frac{1}{\langle s_i \rangle - 1} \left\langle \frac{\partial \log \phi}{\partial h_i} \right\rangle - \frac{1}{\langle s_i \rangle + 1} \left\langle \frac{\partial^2 \log \phi}{\partial h_i^2} \right\rangle \right]. \tag{G.16}$$

In contrast, if we were to do the same thing but take the derivative with respect to the fields in Eq G.11 instead of the natural parameter, we would simply swap the y_i's with the h_i's. This means that we have the reciprocal of the jacobians,

$$(\langle s_i \rangle + 1) \left[(\langle s_i \rangle - 1) \left\langle \frac{\partial \log \phi}{\partial y_i} \right\rangle - (\langle s_i \rangle + 1) \left\langle \frac{\partial^2 \log \phi}{\partial y_i^2} \right\rangle \right]. \tag{G.17}$$

In other words, changing variables gives us a different factor that depends on the mean magnetization of the entries of the FIM. This means that we may expect the overall eigenvalue spectrum to be, at the least, scaled differently. From numerical calculations, we find that the spectrum for observable perturbations to almost always be strictly greater than that for natural perturbations, which may reflect a change in variables.

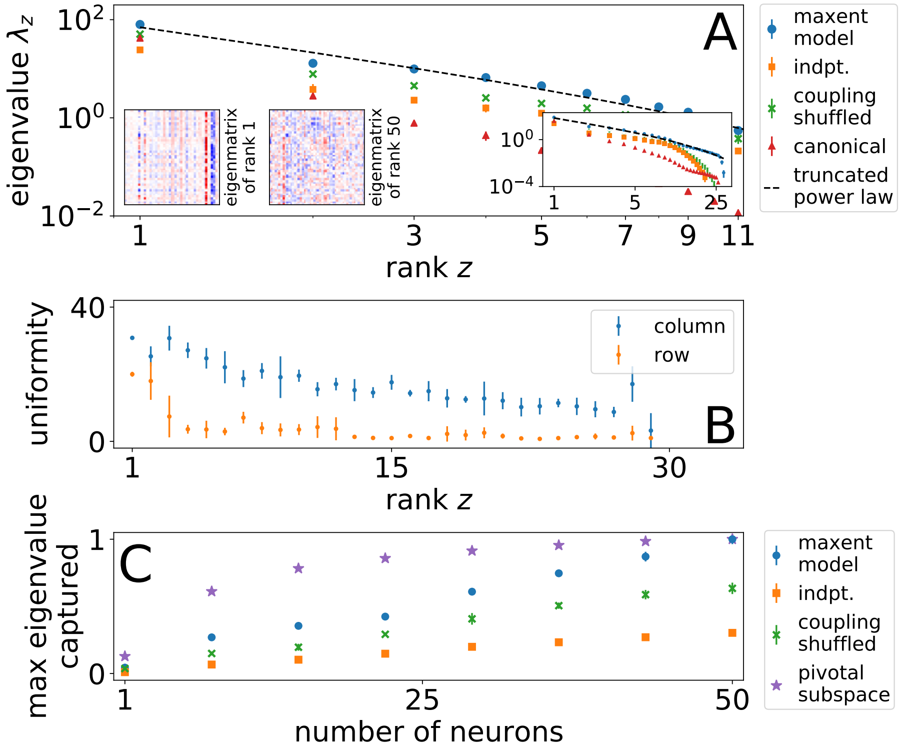

Fig Q. FIM overview for worm experiment 170419 with ϕ_{coarse} instead of ϕ_{fine} as shown in main text in Figure 4.

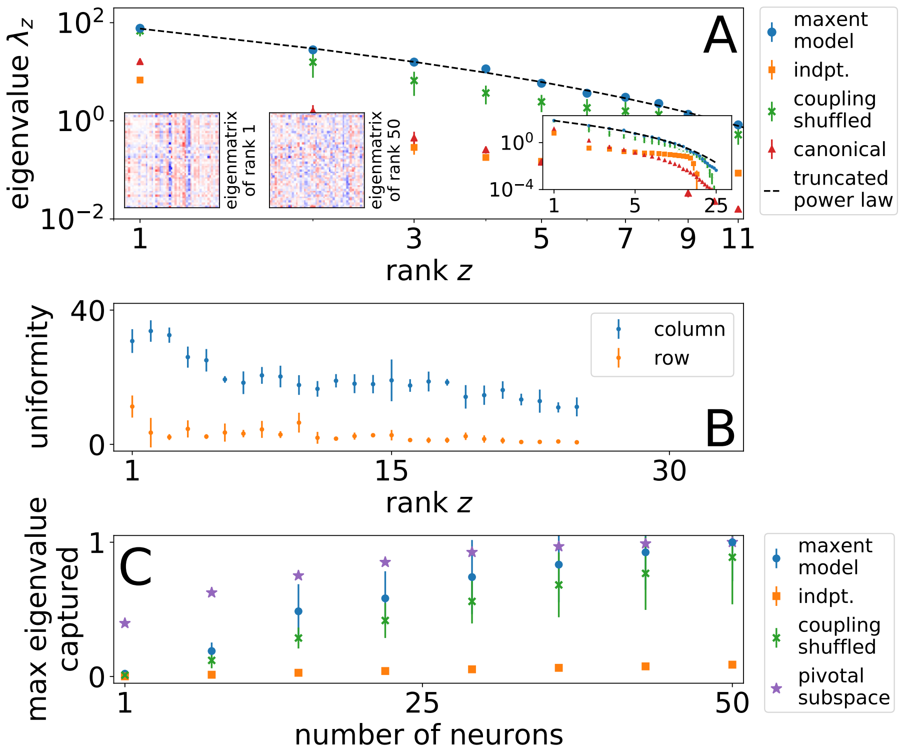

Fig R. FIM overview for worm experiment 154139 with ϕ_{coarse} instead of ϕ_{fine} as shown in main text in Figure 4.

H Data sets and samples

We use the *C. elegans* worm data from Scholz et al. for experiment numbers 170419 and 154139 with bead immobilized worms and published on OSF [23]. For each worm, we consider a limited number of different subsets of $N = 50$ neurons (about the maximum number of neurons for which a pairwise maximum entropy model can be inferred without overfitting), where the neurons have been randomly selected amongst those that have visited all three states at least once. Calcium activity is discretized into up, down, and flat states by its smooth temporal derivatives, using a constant multiplication of the expected standard deviation from a pure white noise to set the cutoff of the flat state. The constant is chosen to be 5 such that in the GFP control worm the same data processing pipeline leads to almost all pairwise mutual information among neurons to be zero. This is the same procedure detailed in reference [16], which also details how the inverse maxent model was solved.

Given the data set we use, we focus on anterior neural activity, which excludes motor neurons that are directly implicated in actuating muscles, and represents about half of the neurons in the mature worm. These neurons are not labeled. Although post-processing may give a rough idea of neural identity, it is of limited reliability. Of these 180 neurons in the brain [25], the data set from Scholz et al. distinguishes about 80 neurons, of which a handful are nearly quiescent. This means that our conclusions are based on a random subsets representing about 1/3 of all neurons from the anterior worm. Since our conclusions are consistent under random subsets of $N = 50$ neurons which also come from an effectively random set in the brain, we do not anticipate our sparseness results to depend on the set of neurons used.

I Experimental implementation

A realization of our thought experiment depends on developments in simultaneous use of recording, perturbation, and computational analysis. The experiment would require tracking time-averaged neural statistics before and during perturbation with the ability to extract simultaneously the discrete state of recorded neurons, a procedure that currently relies on post-experimental analysis to handle noise and changing fluorescence [16]. Furthermore, single-neuron tracking is difficult and may be feasible with immobilized worms, but presents a challenge to perform accurately with freely moving ones. Additionally, it is essential that the nature of the perturbation on membrane voltage be precisely calibrated in order to replicate as closely as possible theoretical clamping, which may require detailed characterization of the particular experimental techniques used. This presents an abbreviated list of some of the experimental difficulties that we foresee for implementing such an experiment that extends on the points made in the discussion.

Though we assume that the perturbation randomly "flips" the neural configuration of the matcher neuron with some small probability at any given moment in time, the perturbation specified in Eq 4 is compatible with variations of the experiment that may be more natural. For example, it may be more natural to force neurons up after a flat state but not directly from a down state. More specifically, it may be important to keep the slope of underlying neural activity from diverging. While such history-dependent clamping is compatible with our formulation that relies only on time averages, experimental comparison might dictate some protocols to be preferable over others in terms of feasibility or correspondence to theoretical predictions.

The timing of the perturbation may also depend on larger-scale dynamics of behavior beyond the calcium ion activity of individual neurons. Since we analyze immobilized worms, we do not consider for the timing of the perturbation worm behavior. Yet, we

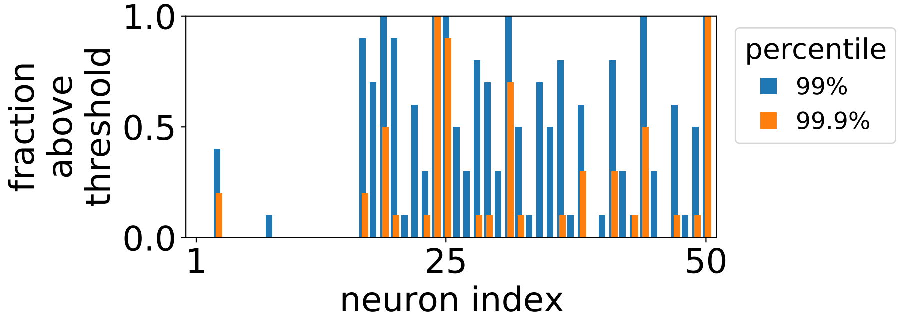

Fig S. Frequency of re-identified pivotal neurons from experiment 154139. See Figure 6 for more details.

know that observations over 10^2 to 10^3 s is commensurate with the timescales over which a typical worm could show reversals and faster headcasts. More generally, biological systems operate across a large hierarchy of timescales. Our model is limited to be an effective representation of interactions over a chosen timescale of interest.

An effective maxent model could be built, for instance, for one particular behavior (say forward motion) over a short timescale, or a different model could represent a mixture over many behaviors over a longer timescale. With this flexibility in mind, an experimentally relevant point is whether or not multiple types of dynamics occur in an observational period. In order to run our proposed protocol, it is important to specify time-averaged neural statistics of interest and to seek out recurrences of the same statistics at later points of observation. When focusing on a particular behavior, a more refined experiment could be to identify different behaviors in real-time in order to isolate measurements and perturbations to the context of a more limited set of behaviors. Thus, while the proposed protocol does not necessarily depend on focusing on a particular range of dynamics, it could be modified to extract pivotal components conditioned on a narrow range of neural activity and behavior. In other words, our modeling approach is flexible with respect to the particular statistics, but that choice must be made consistently throughout the experiment.

Such modifications also assume that behavior could be measured simultaneously with neural activity, which was not possible to do reliably in the experiments we analyzed. Next generation experiments will hopefully reduce instrumental noise in individual neuron measurements with motion, which is a major obstacle for connecting neural activity to behavior. It would then be possible to apply our procedure directly to neural activity and behavior without a proxy such as collective synchrony.

J Modified stochastic mapping

We have assumed that the stochastic encoding relating neural to collective activity does not change, but this is not necessarily the case. To consider this, we characterize the encoding with the fundamental notion of channel capacity, the maximum rate at which information can conveyed by such a mapping given by the mutual information $I[Y;Z] = \sum_{Y,Z} P(Y,Z) \log\left[P(Y,Z)/P(Y)P(Z)\right]$ [26]. A targeted perturbation to the set of states Y corresponds to a new distribution $\tilde{P}(Y) = P(Y)[1 + a_r v_r(Y)]$ giving arbitrary weight a_r to perturbation vector v_r of index r. The perturbation vector v_r may refer to a projection from a single-neuron perturbation or eigenvector considered above. Generally, such a perturbation may change not only the distribution of neural statistics $P(Y)$ but also the mapping $\tilde{P}(Z|Y) = P(Z|Y)[1 + \Delta_{Z|Y}]$, which corresponds

to an adaptive code that changes in response to incoming statistics. Under small perturbations $a_r \ll 1$ and small adaptation $\Delta_{Z|Y}$, the change in the mutual information decomposes into

$$\tilde{I}[Y;Z] - I[Y;Z] = \underbrace{a_r \sum_Y P(Y) v_r(Y) \sum_Z d(Z|Y)}_{\text{direct}} + \underbrace{\sum_Y P(Y) \sum_Z \Delta_{Z|Y} d(Z|Y)}_{\text{adaptive}}, \quad \text{(J.18)}$$

where we have defined $d(Z|Y) \equiv P(Z|Y)\log[P(Z|Y)/P(Z)]$ such that $\sum_Z d(Z|Y)$ is the information gained about Z from observing Y. Eq J.18 contrasts the "direct" result of a perturbation in contrast with an "adaptive" response by the system. Whereas the former term decomposes into the product of the local information geometry of $P(Y)$ and the information content of the mapping $Y \to Z$, the latter depends on system response that modifies the stochastic mapping itself, such as rate limiting or rescaled response [27]. Deviations from the predictions in our perturbative thought experiment might represent the effects of such complications from the properties of the stochastic encoding.

References

1. Barlow HB. Single Units and Sensation: A Neuron Doctrine for Perceptual Psychology? Perception. 1972;1:371–394.
2. Quiroga RQ, Kreiman G, Koch C, Fried I. Sparse but Not 'Grandmother-cell' Coding in the Medial Temporal Lobe. Cell. 2007;12(3):87–91.
3. Daniels BC, Krakauer DC, Flack JC. Sparse Code of Conflict in a Primate Society. Proc Natl Acad Sci USA. 2012;109(35):14259–14264. doi:10.1073/pnas.1203021109.
4. Olshausen BA, Field DJ. Sparse Coding with an Overcomplete Basis Set: A Strategy Employed by V1? Vision Research. 1997;37(23):3311–3325. doi:10.1016/S0042-6989(97)00169-7.
5. Spanne A, Jörntell H. Questioning the Role of Sparse Coding in the Brain. Trends in Neurosciences. 2015;38(7):417–427. doi:10.1016/j.tins.2015.05.005.
6. Beyeler M, Rounds EL, Carlson KD, Dutt N, Krichmar JL. Neural Correlates of Sparse Coding and Dimensionality Reduction. PLoS Comput Biol. 2019;15(6):e1006908. doi:10.1371/journal.pcbi.1006908.
7. Transtrum MK, Qiu P. Model Reduction by Manifold Boundaries. Phys Rev Lett. 2014;113(9):098701. doi:10.1103/PhysRevLett.113.098701.
8. Transtrum MK, Machta BB, Brown KS, Daniels BC, Myers CR, Sethna JP. Perspective: Sloppiness and Emergent Theories in Physics, Biology, and Beyond. J Chem Phys. 2015;143(1):010901. doi:10.1063/1.4923066.
9. Maunsell JH, Van Essen DC. Functional Properties of Neurons in Middle Temporal Visual Area of the Macaque Monkey. I. Selectivity for Stimulus Direction, Speed, and Orientation. Journal of Neurophysiology. 1983;49(5):1127–1147. doi:10.1152/jn.1983.49.5.1127.
10. Wu S, Amari Si, Nakahara H. Population Coding and Decoding in a Neural Field: A Computational Study. Neural Computation. 2002;14(5):999–1026. doi:10.1162/089976602753633367.

11. Cover TM, Thomas JA. Elements of Information Theory. 2nd ed. Hoboken: John Wiley & Sons; 2006.

12. Amari Si. Information Geometry and Its Applications. vol. 194 of Applied Mathematical Sciences. Springer Japan; 2016.

13. Lee ED, Katz DM, Bommarito II MJ, Ginsparg PH. Sensitivity of Collective Outcomes Identifies Pivotal Components. J R Soc Interface. 2020;17(20190873).

14. Nguyen HC, Zecchina R, Berg J. Inverse Statistical Problems: From the Inverse Ising Problem to Data Science. Advances in Physics. 2017;66(3):197–261. doi:10.1080/00018732.2017.1341604.

15. Lee ED, Daniels BC. Convenient Interface to Inverse Ising (ConIII): A Python 3 Package for Solving Ising-Type Maximum Entropy Models. JORS. 2019;7(1):3. doi:10.5334/jors.217.

16. Chen X, Randi F, Leifer AM, Bialek W. Searching for Collective Behavior in a Small Brain. Phys Rev E. 2019;99(5):052418. doi:10.1103/PhysRevE.99.052418.

17. Dudik M, Phillips SJ, Schapire RE. Performance guarantees for regularized maximum entropy density estimation. In: International Conference on Computational Learning Theory. Springer; 2004. p. 472–486.

18. Broderick T, Dudik M, Tkacik G, Schapire RE, Bialek W. Faster Solutions of the Inverse Pairwise Ising Problem. arXiv:07122437 [cond-mat, q-bio]. 2007;.

19. Bargmann CI. Beyond the Connectome: How Neuromodulators Shape Neural Circuits. Bioessays. 2012;34(6):458–465. doi:10.1002/bies.201100185.

20. Bentley B, Branicky R, Barnes CL, Chew YL, Yemini E, Bullmore ET, et al. The Multilayer Connectome of Caenorhabditis Elegans. PLoS Comput Biol. 2016;12(12):e1005283. doi:10.1371/journal.pcbi.1005283.

21. Lu Y, Ahamed T, Mulcahy B, Witvliet D, Guan SA, Hung W, et al.. Extrasynaptic Signaling Enables an Asymmetric Juvenile Motor Circuit to Produce a Symmetric Mature Gait; 2021.

22. Tkačik G, Marre O, Mora T, Amodei D, Berry II MJ, Bialek W. The Simplest Maximum Entropy Model for Collective Behavior in a Neural Network. J Stat Mech. 2013;2013(03):P03011. doi:10.1088/1742-5468/2013/03/P03011.

23. Hallinen KM, Dempsey R, Scholz M, Yu X, Linder A, Randi F, et al. Decoding Locomotion from Population Neural Activity in Moving C. Elegans. eLife. 2021;10:e66135. doi:10.7554/eLife.66135.

24. Nemenman I, Shafee F, Bialek W. Entropy and Inference, Revisited. In: Dietterich TG, Becker S, Ghahramani Z, editors. Advances in Neural Information Processing Systems 14. MIT Press; 2002. p. 471–478.

25. Witvliet D, Mulcahy B, Mitchell JK, Meirovitch Y, Berger DR, Wu Y, et al. Connectomes across Development Reveal Principles of Brain Maturation. Nature. 2021;596(7871):257–261. doi:10.1038/s41586-021-03778-8.

26. Shannon CE. A Mathematical Theory of Communication. Bell Syst Tech J. 1948;27:379–423, 623–656.

27. Brenner N, Bialek W, de Ruyter van Steveninck R. Adaptive Rescaling Maximizes Information Transmission. Neuron. 2000;26(3):695–702. doi:10.1016/S0896-6273(00)81205-2.
