# Supplementary material for: Discovering sparse control strategies in neural activity
Source: PLoS Comput Biol. 2022 May 27;18(5):e1010072. doi: 10.1371/journal.pcbi.1010072 (PMC9140285; doi:10.1371/journal.pcbi.1010072)
Supplement: Attachment — Submitted filename: ref response.pages.pdf [file pcbi.1010072.s003.pdf]

Dear Editor,

Thank you for considering our manuscript at *PLoS Computational Biology*.

We are grateful for the time that the reviewers took to provide detailed and constructive comments. We found many of the points raised to be inspiring and thoughtful, and we hope that our response satisfactorily addresses their questions.

In particular, we were pleased that the referees seemed interested by the “innovative,” “relevant and interesting” substance of the paper and mostly commented on how we could clarify existing sections and presentation. In what follows, we have addressed these main points and have likewise addressed other points one by one.

Overall, we understood the main scientific points raised by the referees to pertain to the clarity, interpretation, and framing of our findings. These points included questions about the generality of our collective measure of neural activity, the modeling approach, and the quality and choice of the data set.

A major part of addressing these comments was a revision in the manuscript’s structure. We have shortened the introduction, shortened the theoretical preface, moved two figures from the appendices while removing one from the introduction, and partitioned the manuscript differently. We now present the extensive theoretical background in its own section with its own overview in the appendices, so that it can be referenced by the reader as necessary.

As part of the reorganization of the introduction and after reflection on Reviewer 3’s comments, we have also altered the title to “Discovering sparse control strategies in neural activity.”

We believe that the resulting manuscript has emerged as a more readable and tighter set of arguments, and we look forward to hearing back.

Sincerely,

Edward D. Lee, PhD
Xiaowen Chen, PhD
Bryan C. Daniels, Professor

Reviewer #1

The authors leverage a maximum entropy (max-ent) model of the neural activity of the nematode C. elegans to probe the sensitivity of some collective neural statistics to experimentally inspired in-silico perturbations. Calcium recordings of neural dynamics are first discretized into 3-states, and a topologically-conserving maximum entropy model is built to match pairwise correlations and mean activity. Given this class of models, the authors choose a fine-grained statistical measure, termed "synchrony" (the number of neurons in each of the 3-states) and probe the sensitivity of this measure to small perturbations to pairs of neurons. The perturbation consists of clamping a matcher neuron to a target neuron with a small probability. Sensitivity of "synchrony" to such perturbations is measured in information-geometric terms through the Fisher Information Matrix (FIM) and its eigenvalues and eigenvectors. Through this methodology, the authors show that the "synchrony" is sensitive to a few principal perturbation modes, with the eigenvalue spectrum exhibiting a power law behavior for a significant number of modes revealing a wide hierarchy of "stiff" directions. In addition, the authors find that the large eigenvalue modes tend to be dominated by a small set of "pivotal" neurons, which tend to consistently increase or decrease the synaptic connections with matcher neurons. Given these findings, the authors argue that control of collective neural statistics is sparse in the number of neurons, while also being non-sloppy in the sense that the eigenvalues of the FIM decay initially as a power law and thus the system does not exhibit a clear separation between sloppy and stiff perturbative eigenmodes.

In summary, I am sympathetic with the general framework of the manuscript. Combining maximum entropy modelling with information-geometric analysis is somewhat innovative, and the authors show that it provides novel insight into the control mechanisms of biological neural networks, in a system that has already been extensively studied. However, besides some concerns regarding the legibility and the structure of the manuscript, I have some deep concerns regarding the validity and the generalizability of the approach that I think need to be addressed before I can recommend publication in this journal.

We thank the reviewer for their encouragement and for their highly detailed critiques. We respond in detail below.

1) The analysis is based on a discretization of the neural dynamics that, while motivated by previous work, might miss important finer scale information. Indeed, unlike the spiking neurons of the mammalian brain, most C. elegans neurons exhibit continuous dynamics, and therefore the 3-state discretization might erase important information. The authors should better motivate this choice, also to help with the generalizability of the approach. Given time series data, how should one discretize before building a max-ent model?

The referee asks whether there is a general way to approach discretization of a time series in the context our approach. While there may be some good rules of thumb as we discuss in our response to part a below, the referee's question of "how should one discretize" is informed by experimental and theoretical practicalities that are difficult to generalize across biological systems.

For example, in the fluorescent data of immobilized worms, the noise appears white and can be distinguished from the signal. Relying on this observation, we apply our smoothing procedure on pure white noise and extract derivatives that correspond to a flat state when they are less than that measured from the noise model. We then confirm this choice of discretization procedure by applying the same data processing pipeline on GFP measurements of neuronal activity in control worms, where we find all neurons are in the flat state most of the time. This discretization method is described in Appendix H.

The minimal description that accounts for inactivity as well as change is the ternary state discretization. This procedure for identifying the "null" state does not provide a general rule but is contingent on the design of the experiment, its resolution, and computational costs.

See below in part a) for text we have added to motivate these choices.

a) I understand that building a maximum entropy model on a system with more states is challenging from a computational perspective, but I believe that this point should be explicitly discussed, especially considering the interpretation of the results.

The referee raises an important question regarding choice of the discretization and how it could be generalized. We now expound in the text:

> Though we might then think to use Ca^{2+} levels as a measure of neural state, we find that fluorescence measurements of absolute levels of calcium are dominated by noise, but it is possible to extract the derivative reliably such that it reflects real correlations in neural activity [48]. Given instrumental limitations that prevent us from measuring the precise continuum dynamics, we focus on a minimal discretization of the time series that still captures the dominant tendencies in the data, coarse-graining each neuron m's derivative as up sm = 1, down sm = −1, flat sm = 0. Whereas a binary discretization would make it impossible to code inactive neurons, a finer discretization leads to exponentially increasing computational costs with the number of states. The optimal procedure should be sensitive to the experimental protocol, but the practical principle of minimal and sufficient description of neural activity leads us to a ternary description of *C. elegans* neural states.

2) It should also be clearly stated that the analysis is focused solely on time-invariant statistics of a 3-state discretization of the neural code. On this point I have a few detailed concerns:

We now explicitly note this and write, "With this discretization procedure, we denote the time-averaged probabilities of being in state *k…*"

(a) Maximum entropy models are typically used to infer the interaction rules that give rise to the observed steady-state distribution (given some underlying dynamics). Therefore, an important assumption underlying the inference of the model is that the system has been observed for a time scale longer than its relaxation time, such that it has clearly reached a steady-state. However, in the analyzed data it is unclear whether the observed statistics truly reflect the steady-state of the system, given that the measurement time, T, is relatively short compared to the transition time scales reported in Scholz et al.

The reviewer is concerned about the large range of timescales on which neural dynamics change and whether or not this is compatible with inferring a fixed probability distribution that characterizes the system in terms of time-averaged statistics.

Given that biological systems typically operate across a large hierarchy of timescales, we view our statistical model as an effective representation of interactions that act over a chosen timescale. An effective maximum entropy model could be built, for instance, for one particular behavior (say forward motion) over a short timescale, or a different model could represent a mixture over many behaviors over a longer timescale. In this view, there is no single "true" steady state of the system. Consequently, we expect the model we infer to be most useful for predicting the results of perturbations over similar timescales, a point we return to below in our answer to 2c.

To clarify this important issue, we now specify in the text, "The solution then defines a minimal interaction network, distinct from the pattern of pairwise correlations, that characterizes dependencies in neural activity across the timescales represented in the data available."

(b) Neurons are inherently dynamical, and thus control mechanisms are also fundamentally dynamical, acting instantaneously on the continuous neural signal. In that sense, the max-ent model used in this manuscript can only probe control at the level of steady-state statistics, and not fine scale control mechanisms.

Certainly. It remains an open theoretical question how control of long-term statistics relates to finer-scale dynamical control. We have added this point explicitly into an endnote for the description of the maxent model, "By definition, the maxent model captures only steady-state statistics and not fine-scale control mechanisms. Later on, we use it to probe control at the level of such statistics."

(c) This is particularly relevant when justifying the use of the maximum-entropy model in an experimental protocol. Real-time perturbations are instantaneous, and measuring their impact through "synchrony" is only possible after a time scale comparable to the time scale used for inferring the maximum entropy model. Relating back to concern a.), it's not obvious that splitting the data in two T/2 segments, for example, would not result in significant changes in the inferred max-ent model, even though there are no apparent external perturbations to the system.

There are two points to address here regarding the effects of splitting the data into two segments and whether or not that would change our results.

The first point is whether or not samples collected for time T/2 would be sufficient to specify the model, assuming that the statistics do not change. This is a problem of model inference. Likely the inferred maxent model would not be the same, but it should preserve the important features (the probabilities of the most probable configurations) considering the sample sizes we have are about 10^3 measurements, which are more than enough to specify the pairwise correlations well.

The second, more relevant point is whether or not two different types of dynamics would occur in the first half versus the second half. This is an important concern and relates to our earlier discussion in point 2a of mixtures of dynamical states. In order to run our proposed protocol, it is important to specify time-averaged neural statistics of interest and to seek out recurrences of the same statistics at later points of observation. In other words, our modeling approach is flexible with respect to the particular statistics, but that choice must be made consistently throughout the experiment. We discuss this further in Appendix I and in the discussion, we have expanded the text:

> Another limitation on implementation is that the duration of the experiment trials and the number of samples needed to estimate a maxent model limit the types of behaviors that could be studied. The experiments we consider last on the order of 10^2 s, and so pairwise correlations can reflect neural activity on the timescale of several correlated behavioral attractors including much faster head casts, bending, and even multiple locomotory reversals and the transitions between them [65,90]. In this sense, perturbations indicate modifications to neural synchrony representative of this mixture and likely not of any single dynamical attractor. In order to run our proposed protocol, it is important to specify time-averaged neural statistics of interest and to seek out recurrences of the same statistics at later points of observation. In other words, our modeling approach is flexible with respect to the particular statistics, but that choice must be made consistently throughout the experiment [91].

3) While it has been shown (Chen et al. PRE 2019 2019;99(5):052418) that max-ent models can accurately approximate higher order correlations between neural states in C. elegans, there is no figure in the current manuscript that shows that that is true in this dataset. The reference given in Chen et al. for the data is a biorxiv preprint (Scholz et al. 2018, https://www.biorxiv.org/content/10.1101/445643v1), which has received contestation by other experts in the field in the comments section of bioRxiv. Indeed, to my knowledge the peer-reviewed version of the referenced manuscript is (Hallinen et al. eLife 2021;10:e66135), and not the reference given by the authors. Thus, I'd recommend that the authors clarify both the source of the data and the capability of the max-ent model to predict higher order statistics in this dataset.

Please see the revised Figure 2 for fit statistics.

The main difference between the two versions of the cited paper is that Hallinen et al. used a blue fluorescence protein to tag neuron AVA. Then, after the whole brain imaging experiment, neuron AVA can be identified, and its trace can be compared with literatures on single neuron dynamics. In Scholz et al., this analysis was not present. The data we analyze in our manuscript (AML32 and AML18 immobilized) is whole brain imaging data for bead-immobilized worms, first published through the data repository of Scholz et al. and specifically referenced in the manuscript (the link to it is here: https://osf.io/79gqk/). We have now cited both manuscripts in the relevant sections.

4) The reasoning behind using subgroups of N=50 neurons should be more clearly explained in the current manuscript. One must resort to reading Chen et al. to know that it is related to overfitting.

We have now clarified this in Appendix H, stating that N=50 was chosen because this is "about the maximum number of neurons for which a pairwise maximum entropy model can be inferred without overfitting."

5) The use of collective 3-state neural statistics as a proxy for behavior is imprecise and unjustified. Behavior is inherently dynamical in nature, exhibiting a continuity of time scales. In C. elegans specifically, behavior ranges from muscle contractions to single body waves, to sequences of body waves that give rise to forward, backward and turning bouts, to sequences of bouts that result in different navigation strategies and so on. On what level is the synchrony measure presented by the authors related to behavior? Specifically, the calcium activity of certain C. elegans neurons has explicitly been associated with the initiation of different bouts, like forward or backward locomotion, but longer time scale sequences of behaviors have yet to be associated with neural dynamics explicitly. In addition, given the inability of the max-ent model to capture neural dynamics, longer time scale sequences of behavior are inherently unattainable in the current approach. If the relationship between the chosen statistic and behavior is not clearly justified, I'd recommend that terms like "neural-behavioral map", as used in the abstract, be removed.

The referee raises an excellent point about our use of a highly simplified notion of behavior falling short of the full complexity of behavior including the multiple timescales on which it acts. This seems to impose a limitation on the kinds of behaviors we could test as "response variables" in an implementation of this protocol.

With the referee's comments in mind, we used the Hallinen et al. dataset to measure the mutual information between our proposed measures of synchrony and the behavioral variables velocity and body curvature. We establish that there is often, though not always, significant mutual information between the two measures of synchrony and each of these two behavioral variables. This is an important point to make in justifying our originally theoretically inspired focus on neural synchrony, and we have added the details to the manuscript accordingly including in Appendix C.

Additionally, we appreciate the referee's point about focusing on the types of behaviors that are commensurate with timescales of the experiments that we are measuring. We now discuss such variation in the discussion at further length.

> Another limitation on implementation is that the duration of the experiment trials and the number of samples needed to estimate a maxent model limit the types of behaviors that could be studied. The experiments we consider last on the order of 10^2 s, and so pairwise correlations can reflect neural activity on the timescale of several correlated behavioral attractors including much faster head casts, bending, and even multiple locomotory reversals and the transitions between them [65,90].

Finally, we have adjusted the abstract accordingly to be clear about the aspirational goals of our protocol in exploring the neural-behavior map:

> Using *C. elegans* as an example, we propose a protocol for systematic perturbation of neural states that limits experimental complexity and **could eventually help** characterize collective aspects of the neural-behavioral map. We consider experimentally motivated small perturbations — ones that are most likely to preserve natural dynamics and are closer to internal control mechanisms — to neural states and their impact on collective neural activity.

6) Related to both points 3) and 5), the authors provide no justification for the use of a dataset with only two recordings on immobilized worms, even though the data provided by

Hallinen et al. includes also other experimental protocols, including recordings in freely moving worms. What is the reason for excluding this data? This is especially puzzling given the authors' claim that the presented approach can be generalizable to other "measures of internal states" including behavioral output. Could these statements be made precise? After all, simultaneous measurements of neural and behavioral dynamics are available, and I'd be curious to see how to translate this approach to behavioral dynamics.

While mobile worm data exists for both the Scholz and Hallinen data sets, there are practical limitations on simultaneous measurement of neural activity and behavior. The neural activity measured in these datasets exhibits substantial spurious correlations between pairs of neurons due to the difficulty of removing motion artifacts in the case of moving worms. These motion artifacts are small enough to provide behavior prediction as in Hallinen et al., but they are too large for derivative-based discretization of neuronal signals; for example, the same data processing pipeline applied to a moving GFP control worm will show that these neurons are "active." This suggests that at the current stage of the data, the instrumental noise is too large to perform similar analysis for moving worms. For this reason, we used the cleaner neural data available for immobilized worms.

7) The use of the synchrony statistic should be better justified. In what sense is this a complete enough measure of collective neural states and, more importantly, behavior? This is especially important given that all the results of the manuscript depend on this statistic.

In addition to the below responses, we have largely addressed this above in the response to point 5.

a) How much more computationally expensive is it to include state identities into synchrony statistic?

While this would increase the number of states nominally by a factor of 6 (since there are 6 ways to assign 3 states to 3 bins), it would be orders of magnitude more expensive to obtain the same amount of accuracy for the probability distribution from Monte Carlo sampling. This is because we would need (in a good case) 36 times more samples to obtain a similarly good estimate of the probabilities of each synchrony outcome and other loops in the code would need to be run 6 more times. Our calculations currently require substantial supercomputer time, such that increasing the time by a factor of 100 is currently infeasible.

b) How justified is the connection to the concept of sloppiness? Sloppiness is studied with respect to the likelihood function of a set of parameters given the data (Transtrum, J Chem Phys 2015;143(1):010901), which is not the same as choosing a test statistic and examining how it varies with respect to perturbations. Why is the FIM not computed w.r.t the likelihood function?

As the referee notes, in the sloppiness literature the FIM is computed by varying model parameters in order to investigate how quickly the likelihood of the data changes. Here, we vary model parameters in order to determine how responsive collective activity is to systematic perturbation. In both cases, information geometry defines the duality between observable statistics and the parameters of the model.

This means that, if we assume that the observable quantity is the collective test statistic and if our model is an exact reproduction of the observed statistic, the FIM computed with respect to the likelihood function is exactly the same as that using our model test statistic. It is in this sense that we make a connection to the sloppiness literature, which we view as being fundamentally focused on characterizing the mapping from "microscopic" parameters to "macroscopic" outcomes, common to both of these interpretations.

8) The authors make no attempt to connect to other modelling paradigms of collective neural activity in C. elegans, even though there are several examples that even connect to the underlying neurobiological control. Here's a few examples, although there are others:
i) Brennan et al. eLife 2019;8:e46814

ii) Morrison et al., Front Comput Neurosci. 2021 Jan 22;14:616639
iii) Fieseler et al., J. R. Soc. Interface.1720200459

Thank you for bringing these to our attention. We have read the manuscripts and have incorporated them into a paragraph in the introduction about sparse neural and behavioral dynamics. As the referee notes, there are other paradigms for modeling neural activity including detailed biophysical models of the connectome (Liu et al., 2017) and stochastic linear models (Linderman et al., 2019). A number of modeling approaches are nicely organized in Morrison et al. (2021), but not all of them are directly pertinent to the discussion. The relevant paragraph in the introduction is

> In the model organism *Caenorhabdits elegans*, the hypothesis that neural control is sparse is supported by observations that both neural and behavioral dynamics live on sparse manifolds. Neural activity displays collective modes originating from circuit components or global encoding of information that effectively reduces its dimensionality [5, 17, 48, 50]. Complementing this, worm posture is low-dimensional, and four eigenmodes capture nearly all the variance in shape space [42,51]. More recent work combines the two types of measurement to show that dynamical embedding of worm locomotion along dimensions of neural activity extracts a few principal neural dimensions that capture transitions between behaviors [52,53]. In some cases, neural modes are even predictive of longer-term behavioral regularities [54]. Though neural signals may not simply reflect behavioral control, these findings support the hypothesis that common mechanisms of neural control may underlie neural-behavioral similarities between individuals. Inspired by signatures of sparseness in neural-behavioral mapping, we consider how sparseness in collective neural activity responds to changes in individual neurons as a step towards considering behavior.

9) The Fisher Information Matrix computed in the manuscript measures the sensitivity of the chosen test statistic, to perturbations that are reflected in the changing of model parameters. In that sense, it seems to be specific both to the model class and the chosen statistics. How generalizable is this? Can this FIM analysis be reproduced using other modelling paradigms?

Our framework is generalizable and can be used with other modeling frameworks. One of the major innovations of our framework is the use of observable perturbations, which can be implemented in a precisely comparable way in any model, regardless of its structure. In other words, any model that allows us to compute statistics of neural synchrony can in principle be used directly in our framework.

In this work, we focus on clearly laying out this framework as opposed to exploring how the results may depend on competing neural models and collective statistics. We now explicitly mention this as a place for future work in the discussion:

> In the case of Boltzmann-type models, the relationship between observables and model parameters can be made exact for small perturbations. On the other hand, other neural models may be able to more faithfully capture temporal dynamics or consider additional collective statistics that could be incorporated in future work. Thus, our formulation is a theoretically extensible and experimentally tractable framework for predicting the effects of perturbations.

To clarify our use of observable perturbations as a significant shift in the way we think about parameters in phenomenological neural models, we have extended our discussion in “Mapping perturbations from experiment to simulation”:

> The replacement rule maps perturbations on observed statistics to system sensitivity instead of those on model parameters as is often the first instinct for theorists (see Appendix F). In the latter formulation, the parameters of the maxent model such as fields and couplings form the basis for “natural” or “canonical” perturbations [75]. This perspective adheres to the physical intuition that the parameters in the energy function reflect latent physical interactions. When the pairwise maxent model instead serves as a phenomenological approximation of the underlying physical interactions, a natural perturbation may be mediated by unknown

> parameters instead of fields and couplings [56,76]. This obscures the intuition that changing fields should alter the individual neuron biases while couplings should alter the tendencies of pairs to agree.
>
> Partly for this reason, the straightforward model perturbation to couplings in Figure 3B becomes much more complex when translated into its reciprocal experiment in Figure 3A. We show an example of a set of neural perturbations in panel C, indicating roughly uniform perturbations to three dominant neurons, that is more complex in the reciprocal space of fields and couplings in panels D and E, respectively. Again reflecting this basic principle, a trivial coupling perturbation in panel G is complex in the space of matcher-target perturbations in panel F. Additionally, observable perturbations do not depend on the model used but are preserved regardless of how a model is parameterized, ensuring consistency when our framework is extended to other model classes. Thus, defining perturbations in observable terms as in Eq 4 allows for easier experimental interpretation and for consistent comparison of inferred models [47].

Besides these more general concerns, I also have an extensive list of more technical comments:

1) There is a typo on line 16: neruons should read neurons.

This has been fixed.

2) In the paragraph starting in line 18, environmental conditions should be included as an important barrier to the connection between global neural state and behavior. Indeed, this study uses experiments in which worms are immobilized and drugged, which severely impairs the mapping between neurons and behavior.

Given the reorganization of our manuscript, we have placed such discussion in a couple other locations. We now discuss the role of environment in the discussion,

> Calcium levels may indicate not only behavioral actuation and modulation but also proprioception, efference copies, or sensory information. They may depend strongly on environmental constraints such as bead immobilization here.

3) I really cannot see the relevance of the analogy with sensory processing (line 30) in the context of the neural-behavioral map, behavior is very different from perception. Can you clarify this point?

We mean that the mathematical formulation relating stochastic input with output is applicable to both situations. However, this does not imply that same types of encoding apply in both perception and behavior. We have altered the corresponding paragraph, now the first in Appendix A, to be more clear about the relation:

> The question of control is closely linked to how neural states map to behavior. When the mapping is not deterministic, this is a problem of stochastic mapping of the behavioral state Z to the neural state Y as pictured in Figure A.7. A simple example comes from the context of sensory coding, where "grandmother" neurons represent increasingly complex patterns of sensory stimuli as information is funneled up a hierarchy [41,107]. In this case, the mapping between Y and Z is precise.

4) The statement in line 43 starting with "In the formulation" is unclear and grammatically incomplete.

This sentence has been corrected: "In sparse coding, a basis set of neurons are extracted from measuring neural response to visual stimuli, where neural activation represents abstract image features such as edges, corners, and contours."

5) Reference 48 in line 104 is not very precise in the context of behavioral sequences, specialy for C. elegans. While discrete behavioral states are defined in ref. 48, no explicit

temporal sequences of behavior are described. In the context of C. elegans other references would be more suitable, e.g. (Gomez-Marin et al., J. R. Soc. Interface 13: 20160466) or more recently (Costa et al., arXiv:2105.12811v2).

We have changed the reference accordingly.

6) How exactly are the states "flat", "increasing", "decreasing" defined from the calcium signals? Information about the discretization of the data should be explicitly included in the methods. The reader shouldn't have to resort to other references for such important information.

This information is now in Appendix H.

7) In line 146, reference to figures A6 and A7 is unjustified. Where is there a figure to support the fact that accounting for pairwise correlations is sufficient to capture higher-order correlations? Also, at this stage the statistic phi_coarse is not defined and perhaps should be defined in the figure caption.

The panel C in Figure 2 now shows phi_coarse(k), which is a higher-order statistic since it is the average of kth-order correlation functions. It is defined in the text as "probability that a plurality of n neurons coincide phi_coarse(n)." We show that the pairwise model does substantially better than the independent model and is quite close to the data.

This is also the case for phi_fine, another measure of higher-order correlations, as we now show in an additional panel D.

8) There is no reference in the text of the manuscript to Fig.3. Also, it is not specified whether phi_fine or phi_coarse are used in Figs.3C,D,E,F,G. In addition, Fig 3D is very hard to read.

We now refer to Fig. 3 in the section "Mapping perturbations from experiment to simulation". We have clarified that phi_fine is used in this figure. We have simplified Figure 3D and made it larger to make it easier to read.

9) The section about the different types of perturbations starting in line 188 should be made clearer and perhaps there should be a reference to Appendix D.

We have restructured the paragraph and included a reference to what is now Appendix F. The revised text has been cited as the response to major comment 9.

10) The reference to Fig. C.10 in line 234 is challenging to understand as the exponents have not been defined yet.

We have removed this reference.

11) In lines 236 and 237, it's not at all clear where the Z_max values are coming from, and Fig. E.19 is not clarifying that. There should be more guidance to the figure or in the figure captions to make these points clearer.

We have revised the sentence to emphasize the stated definition: "Across all cases, we expect a hard cutoff at rank $Z_{max} = 234$, the dimensionality of the synchrony distribution ϕ_{fine}."

12) In the paragraph before Eq.(8), how are the diagonal blocks exactly defined? Is it possible to show a figure of these matrices and how the blocks are identified?

The diagonal blocks are defined as the set of all outgoing perturbations from matcher neuron *m* to all neighbors *t*. Given the large size of the matrix (1225 x 1225), we do not think it would be much helpful to show the matrix. Since the definition is already stated in the manuscript, we have included a citation to previous work, where an example of such a matrix is shown as is copied and pasted below:

13) In Eq.(8), how exactly are the eigenvectors normalized?

(C) Sensitivity matrix

$$F_{\mathrm{x'y'xy}} = \lim_{\epsilon\to 0} \frac{2}{\epsilon^2} D_{\mathrm{KL}}\left[q_J || \tilde{q}_{\tilde{J}}\right]$$

$$\tilde{J} - J = \Delta_{\mathrm{xy}} J(\epsilon) + \Delta_{\mathrm{x'y'}} J(\epsilon)$$

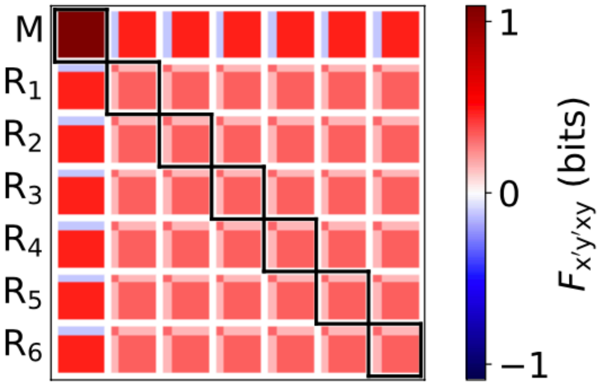

The eigenvectors are normalized to unit length, which is the standard normalization, now specified as having “unit norm.”

14) The shuffle models are not clearly explained in the text, and so their relevance becomes difficult to judge. What is the point of comparing with the different shuffles? There should be a section explaining these in detail and their relevance. In particular, the shuffled couplings control is confusing as it seems to only shuffle the neuron indices but preserving everything else. What’s the point?

Since we started with a minimal model that was intended to recover the topology of neural interactions, we use a shuffling of couplings to test if the topology matters for the patterns we see. This we now state in the text: “couplings randomly shuffled between all pairs of neurons (which preserves the distribution of couplings but not the topology of the interaction network).”

Following the referee’s suggestion, we emphasize the point of such a shuffling by rewriting:

> The shuffled couplings model, however, is much closer than the others to the scaling for the pairwise maxent model of the data, suggesting that the topology of the interaction structure is not primarily determining sensitivity but that *sensitivity depends on statistical properties of the coupling distribution*. This is evocative of theoretical results analyzing the stability of neural networks [81].

15) How surprising is it that the pivotal neurons fluctuate across random subsets, given the fact that the statistic that is used to compute the FIM is independent of state and neuron identity?

We would expect that the exchange symmetries permitted by the synchrony statistics would make it more likely that multiple neurons end up having the same effect, not that particular neurons would be singled out as having a larger effect. In this sense, the symmetry of the collective statistic highlights that some property of the local interaction network and biases must distinguish pivotal neurons from others.

While pivotal neurons fluctuate because of stochasticity in the random sample, this also means that there are several, competing highly sensitive modes, and we show that they are all characterized by pivotal neurons regardless of the many symmetries permitted by synchrony.

a) Relatedly, it should be possible to reliably obtain neuron identities, even if only from a subset of neurons. Certain neurons are known to be important for initiating and maintaining certain behaviors (e.g. AVA), where are these in your analysis? Do the most consistent pivotal neurons identified in your approach correspond to known important neurons for C. elegans behavior? It would be very interesting if you could establish any testable predictions regarding the underlying neurobiology.

Indeed, this would be extremely interesting. Unfortunately, while we did inquire about this, one of the authors of Scholz et al. indicated that neuron identification was unreliable.

16) In Fig. 4A, the inset matrices are hard to see. I'm recommend expanding them.

We aim to show the presence and absence of vertical striations and not the matrix elements. We clarify this in the caption. Example matrices are in Figure C.10.

17) The discussion paragraph starting in line 320 is also confusing. Doesn't shuffling the interactions effectively give neurons different roles?

Yes. We now discuss this point more extensively in a following paragraph:

> The collective properties require neural heterogeneity. Since identical neurons would imply uniform eigenvectors, variation in each neuron's local interaction network and bias is responsible for the emergence of pivotal neurons. By shuffling the inferred interaction network, we verify that these features seem to be a result of the distribution and magnitude of interactions but not the specific topology. In this sense, the neurons do not need to be labeled differently, but the collection of local interactions and biases is responsible for the statistical properties of the FIM, echoing findings about statistical properties that make neural networks stable [81].
>
> At first glance, this seems to be at odds with the consensus that neurons involved in motor circuits play fixed roles such as AVAs in reversals [4]. It is not. We focus on collective synchrony as a simple proxy for behavior, and interchangeability of pivotal neurons may stem from the way that synchrony treats each neuron equally. By definition, it cannot distinguish between neurons with respect to a particular behavior. If we instead imagine searching for controllers of specific behaviors, this symmetry is likely lost such that control may be localized in fixed neurons. It remains to future work to reconcile our result about a statistically robust property of collective neural activity and its functional implications for sparse behavioral control.

18) In the second paragraph of Appendix A there is a typo. Before references [90,91], "of" in "of the likelihood" should be removed.

This has been corrected.

19) It is difficult to see, comparing Fig. C14 with Fig. C11, that the distinction of pivotal neurons from uniformities with the MCH model is not as consistent as with the topological max-ent model introduced previously. Can this be explicitly quantified?

These figures are now E.13 and E.16.

We agree that there is indication of pivotal neurons even with the MCH solution. Our observation is that the difference between the two curves is not as consistently large. This can be shown by integrating the difference between the two curves, but since the particular quantity has no particular importance, we do not show it.

20) In Fig A.6C, the figure caption for the inset seems to be wrong.

We have corrected this. The description of the inset was wrong.

21) The results of Fig.B.8 are insufficient to show that the eigenvectors of the inferred FIMs are robust to the sample size K. In fact, there seem to be significant differences in the secondary eigenvector between K=10^5 and K=10^6. Such differences should be better quantified, and this analysis should be done across multiple samples and not just one example.

The referee points out that there is significant variability between Monte Carlo samples in the structure of the eigenvector depending on the size of the sample (now Figure D.10). These differences are not relevant for the conclusions we reach, which have to do with the statistics of such matrices. We mention in the discussion, "Instead of specific pivotal neurons, it is the collective properties of localized eigenvectors and scaling in sensitivity that are preserved." As we show in the top panel of Figure D.10, the eigenvalue spectrum is preserved. In Figure E.13, we

show that the separation in row and column uniformities is preserved across Monte Carlo samples and neural subsets.

22) There is a collection of typos in the first sentence of Appendix C.

We have fixed these.

23) Across the supplementary figures subsets A,B,C and D are used. How exactly are these subsets defined?

These are results from subsets of N=50 neurons following the steps of previous work from Chen et al. We specify in Appendix H "Data sets and samples,"

> For each worm, we consider a limited number of different subsets of N=50 neurons (about the maximum number of neurons for which a pairwise maximum entropy model can be inferred without overfitting), where the neurons have been randomly selected amongst those that have visited all three states at least once.

24) There is a typo in the last sentence of page 24: "and" in "consider and the fine" should be removed.

This is not a typo, but it may be clearer to remove "the" before "fine." We have gone further by rewriting, "Across the maxent models we consider and both measures of synchrony, we consistently find that the decay exponent is faster for natural perturbations, in alignment with our results for pairwise perturbations."

25) Some supplementary figures have been given the wrong labels. For instance, Fig. E.17 pertains to Appendix D but has been labelled as if belonging to Appendix E, and Figures G have nothing to do with Appendix G. The labelling is confusing.

We have placed these figures in the right sections.

26) "Furthermore" is repeated in two subsequent sentences in the first paragraph of Appendix G.

We have fixed this.

Reviewer #2

Summary of the paper:
The authors of the manuscript “Discovering sparse control strategies in C. elegans” describe an analytical perturbation protocol that is potentially experimentally feasible and should yield insight into the neurons that are most relevant to behavior. They use a (published) maximum entropy model extracted from the measured neuronal activity of C. elegans to test the impact of their pairwise perturbations in this system, finding that some specific ‘pivotal’ neurons are very sensitive to perturbation, possibly indicating that these are also relevant for behavior.

Review summary:
While I think the work itself is interesting and a relevant addition to the field, adding rigorous theoretical underpinnings to perturbation experiments that are now feasible or at least within reach, I have some major concerns about this paper which I detail below. Foremost, these are concerned with the presentation and context of this work, rather than the theoretical results. I therefore believe these are addressable by a major revision and editing for clarity.

We are pleased to read that the reviewer found the work worthwhile to publish pending manuscript restructuring. We have reorganized the introduction to separate its contextual framing from the theoretical background, which we now present in a separate section.

Major issues:
There are some inconsistencies in the framing of the paper: the authors claim in the introduction and the abstract that this perturbation protocol is “(...) for systematic perturbation of neural states that limits experimental complexity but still characterizes collective aspects of the neural-behavioral map.”. The introduction refrains from considering any of the non-feedforward nature of neural systems where not all neural signals are directly mapping to behavior. Representations of behavior might be due to motor commands, or sensory-motor transformation. But they might also reflect proprioception or efference copies. The connection from neural activity to behavior and the concrete impact of this work needs to be better explained, especially since the maxent model is based on calcium imaging data.

The referee raises the point that calcium activity may reflect more than a feedforward connection to behavior. Indeed, calcium activity may indicate something that can or cannot causally change behavior. Inference of causal relationships is a general limitation of any study that uses correlations either in neural activity as we do or when relating simultaneous measurements of neural activity and behavior. Thus, our claims based on our maximum entropy model present testable predictions about how perturbations may alter collective activity and eventually behavior.

We had hoped to make our assumptions clear with our discussion of stochastic mapping (formerly in the introduction). Since this point did not come through, we now mention this briefly in the introduction and discuss further in the conclusion:

> An important caveat regarding our predictions is that the model is based on neural activity that may reflect other types of activity [40]. Calcium levels may indicate not only behavioral actuation and modulation but also proprioception, efference copies, or sensory information. They may depend strongly on environmental constraints such as bead immobilization here. Then, the interactions that we recover between neurons would confound physical and apparent interactions. As a result, a statistical connection from in silico individual neuron to collective synchrony may not indicate a causal pathway. Though this is a problem common to studies that rely solely on measurement data, previous work shows that maximum entropy models can in some cases identify real physical interactions from correlations [86]. A definitive answer, in any case, needs a perturbative experiment, and our formulation provides a set of predictions that can be directly checked.

A key relevant citation for high-throughput optogenetics in C. elegans is missing: https://doi.org/10.1038/s41592-018-0233-6 which also finds a small number of locomotion-relevant neurons in the worm

We thank the referee for bringing this relevant and interesting paper to our attention. We have now included this paper in our citations.

The introduction mixes organisms, techniques and results across a number of (very different) organisms to support their points. For example in ln.13-17, and especially ln. 58 where Phineas Gage and worms find themselves in the same citation. The motivation could be strengthened significantly by selecting citations from the same or similar organisms that are technically available for these measurements or alternatively expanding the discussion of the other results substantially and how these affect the interpretation with respect to the proposed perturbation approach.

We have narrowed the scope of the introduction accordingly and now more clearly distinguish between the generalizable questions and specific application to the model organism *C. elegans*. In the discussion, we elaborate on extensions to other systems:

> Our procedure is general enough to be applied to other neural systems and collective behavior in systems biology. Experimentally, our method requires measuring and making small perturbations to internal states at the level of individual components, along with the simultaneous measurement of a collective state or output behavior. Theoretically, it relies on a statistical modeling approach that assumes a discrete, time-independent description. One could imagine adapting the method to internal states such as hormone concentration, gene expression, or other experimentally accessible quantities [92–97] and to output behavior including morphological descriptors [42,98,99], a stereotyped behavioral sequence [51,100,101], or developmental outcomes. Of these possibilities, the most straightforward adaptation would be to other model neural systems such as cortical cell cultures or muscle neurons involved in *Drosophila* flight [102]. Although neurons in these systems show discrete action potentials instead of graded activity, the standard mapping to binary states could lead to comparable results. Similar modeling approaches to the aforementioned systems, such as by discretizing levels of gene expression, could lay the basis for further extensions of the approach. In this sense, our work provides a generalizable theoretical framework that also incorporates specific perturbative predictions that could be tested experimentally across multiple biological systems.

There exist a number of similar, published datasets from other groups (e.g. from the Zimmer lab). Do the results generalize to datasets obtained with slightly different imaging modalities? If not, why?

We expect our results to generalize, but there may be subtleties with respect to the way that raw fluorescence levels are normalized and then discretized. Given the importance of other experimental subtleties (not least missing or corrupted data points or reliability of imputed derivatives) we have decided to work with a data set that we are well familiar with.

Clarity of text and presentation: Panels and one figure seem to not be cited in the main text (e.g., Figure 3, 4B). It is also a bit confusing when large sections of the text refer to figures that are relegated to the appendix, this is mostly the case in the section about the Eigenvalue decay statistics. I think the work could benefit by a thorough editing for clarity in text and visual presentation. The appendix is clearly written and more approachable than the main text, and the main text would benefit from a similar tone (e.g Appendix A and C are particularly well done). I wonder if the authors would consider compressing the main text even more and focusing on the main argument of the perturbation strategy and the results

for the C. elegans datasets, to make this manuscript more easily readable and a useful resource for different readers.

This comment about improving clarity is echoed by the other reviewers. We have addressed this by significantly restructuring the manuscript. We have shortened the introduction, added two figures to the main text that had been referenced (but directed the reader to the appendices), and separated out a theoretical implementation section. We aim to make it easier for the reader to reference the relevant sections and see the flow of the manuscript.

In the calculation often a subset of N=50 neurons is used, when the nervous system of the worm comprises 302 neurons. Does the fact that in this subset some coupling are missing affect the conclusions, especially the conclusion about sparse control? If not, then do we learn something about an efficient experiment where one only needs to observe a few neurons?

The referee asks if our conclusions would change having measured more than the 50 neurons that we use as subsets for our analysis. We note that we focus on subsets of N=50 neurons because this is the maximum number that the pairwise maxent model can match without overfitting given the duration of the time series (Chen et al., 2019).

One important restriction is that we focus on anterior neural activity, which excludes motor neurons that are directly implicated in actuating muscles and represents about half of the neurons in the mature worm. Of these 180 neurons in the brain (Witvliet et al., 2021), the data set from Scholz et al. distinguishes about 80 neurons, of which a handful are nearly quiescent. This means that our conclusions are based on experimentally random subsets representing about 1/3 of all brain neurons. (We have added some of these details to Appendix H). Since our conclusions are consistent for multiple random subsets of N=50 neurons which come from an effectively random set of neurons in the brain, we do not anticipate our sparseness results to depend strongly on the number of neurons measured.

Indeed, the robustness of our results to neuron identity suggests to us that measuring fewer neurons could be sufficient for predicting sparse control mechanisms, at least for controlling highly symmetric collective variables such as the ones we test. If we instead imagine searching for controllers of specific behaviors, this symmetry is likely lost, such that control may be localized in particular neurons. In this case, leaving neurons unmeasured risks not finding some particular important neurons. These are important practical matters for future experiments. We now include in the discussion,

> At first glance, this seems to be at odds with the consensus that neurons involved in motor circuits play fixed roles such as AVAs in reversals [4]. It is not. We focus on collective synchrony as a simple proxy for behavior, and interchangeability of pivotal neurons may stem from the way that synchrony treats each neuron equally. By definition, it cannot distinguish between neurons with respect to a particular behavior. If we instead imagine searching for controllers of specific behaviors, this symmetry is likely lost such that control may be localized in fixed neurons. It remains to future work to reconcile our result about a statistically robust property of collective neural activity and its functional implications for sparse behavioral control.

I am curious to know if the fact that the model is derived from calcium imaging data changes the interpretation of the model at all. It seems that calcium can detect neural activity easier than suppression such that there is a fundamental asymmetry in the underlying data. How does this impact the ability to detect certain couplings, and relatedly the column uniformity in Fig.4?

The referee points out that calcium imaging data may constitute a fundamental bias our approach because we are measuring calcium levels, which are a positive quantity.

We first note that our formulation allows for at least a simple representation of both activation and suppression since we are measuring the change in calcium activity over time. Indeed, we find that both increase and decrease in calcium is captured.

We also recover negative interactions in the model, indicating that we can capture inhibitory relations from the data (Figure 3).

If the referee means that calcium can decay slower than it rises, then this is an inherent bias of the system that we believe *should* be reflected in the data and in our model.

I am a bit confused by the following statement: In 285-286: "In contrast with the typical notion that the identities of particular neurons are essential, pivotal neurons fluctuate between subsets and random samples as shown in Figure 5." This seems to contradict much of the literature in which neurons do play distinct roles in behavior and these have been identified in the worm e.g. AVAs roles in reversals. It would be helpful for the reader to unpack this and similar statements about the sloppy-sparse control and put them in the context of the real, biological network. This would add to the discussion.

We agree that the relationship between neuron identity and our pivotal neurons is a subtle point that deserves more attention, especially as it is central to our conclusions. We now discuss this at further length in the discussion as is copied below (some of which was mentioned above):

> As an in silico realization of the protocol, we use perturbations that mimic internal neural coordination and calculate their impact on collective synchrony. In a minimal model, we find that dominant perturbative modes do not tend to be distributed amongst many neurons but are localized into pivotal ones. The concentration of sensitivity in a few neurons is analogous to the presence of driver neurons, modulation of which drives the system from one configuration to another [83–85]. Interestingly, we find that pivotal neuron identities fluctuate across subsets and Monte Carlo samples used to estimate the entries of the FIM. Instead of specific pivotal neurons, it is the collective properties of localized eigenvectors and scaling in sensitivity that are preserved.
>
> The collective properties require neural heterogeneity. Since identical neurons would imply uniform eigenvectors, variation in each neuron's local interaction network and bias is responsible for the emergence of pivotal neurons. By shuffling the inferred interaction network, we verify that these features seem to be a result of the distribution and magnitude of interactions but not the specific topology. In this sense, the neurons do not need to be labeled differently, but the collection of local interactions and biases is responsible for the statistical properties of the FIM, echoing findings about statistical properties that make neural networks stable [81].
>
> At first glance, this seems to be at odds with the consensus that neurons involved in motor circuits play fixed roles such as AVAs in reversals [4]. It is not. We focus on collective synchrony as a simple proxy for behavior, and interchangeability of pivotal neurons may stem from the way that synchrony treats each neuron equally. By definition, it cannot distinguish between neurons with respect to a particular behavior. If we instead imagine searching for controllers of specific behaviors, this symmetry is likely lost such that control may be localized in fixed neurons. It remains to future work to reconcile our result about a statistically robust property of collective neural activity and its functional implications for sparse behavioral control.

Minor:

Paragraph ln. 93-106 missing citation 10.1016/j.cell.2020.12.012

We have added this.

Citation [48] should instead cite one or more of the many C. elegans behavioral papers that are relevant here

We have added citations for other measures of internal states such as hormone concentration (in the context of development), gene expression, and locomotion.

Panel 4C: the maxent model data is barely visible

We have shifted the markers slightly to make them more visible.

Check panel labelings 4

We have revised the caption.

Appendix C '...turning them up, for a particular neuron is effective'...effective in what way?

We have rewritten this to state, "This is a feature of localized control because it means that turning off all local synaptic connections, or turning them up in a way that makes the matcher more similar to all other neurons, is effective."

Reviewer #3

The authors develop a theoretical framework using perturbation experiments to discover the neurons in a nervous system that induce the most change in the behavior of the system. They apply their methodology to a minimal model of C. elegans neural activity, and they find the model is most sensitive to a few principal perturbative modes.

One of the primary contributions of this work is the way in which extends earlier work on "sloppy" models. Whereas the original work primarily relies on an analysis of the curvature of the manifold from the dynamical description of the system, this work estimates the curvature through local perturbations. This is of practical use to neuroscientists who can only perturb their model living organisms.

My main issue with this paper is that it is hard to follow. This is partly because the approach is itself relatively sophisticated. But part of the difficulty is due to the writing and the organization.

We appreciate the reviewer's comments, which echo the others, and we have significantly reorganized the manuscript and tightened up the language to improve legibility. We hope that these changes have addressed the reviewer's concerns.

According to the title, the expectation is that there is a certain focus on learning something specific about C. elegans. However, the C. elegans model as an example system diverts from the real focus of the contribution, which is the framework. The paper's main contribution does not seem to be about the discovery of sparse control strategies in C. elegans. Instead, the paper's central contribution seems to be about the framework for discovering the neural components that affect behavior, and therefore are likely to be involved in the control of the nervous system.

After reflection, we agree with the reviewer that our contributions could be more clearly expressed by separating out the connection to *C. elegans*, which we use as an example but is not the main focus of the study. We have changed the title accordingly to "Discovering sparse control strategies in neural activity." The *C. elegans* example is still important in providing a realistic case study to demonstrate our methods (in a way that a purely theoretical perspective would fail to illustrate—see below), so we have kept it in the abstract and introduction.

Presumably the choice of a C. elegans model was to provide useful predictions for experimentalists. However, the predictions are not clear, particularly in relation to understanding the neural basis of C. elegans behavior. And unfortunately, the choice of C. elegans model leaves the validity of the framework untested. Given that the focus was on showing the utility of their novel perturbative approach, a simple toy neural-behavioral model, where the ground-truth would be known and tractable, would have made the approach both more convincing, and more easily interpretable.

My suggestion would be to improve the clarity of the contribution by either making the focus more clearly on C. elegans and elaborating more succinctly on the predictions, or to focus more concretely on the framework and to provide first a toy model that illustrates the usefulness and validity of the approach, before applying it to data from C. elegans.

The referee notes that there are two major points in the paper, theoretical development of an experimental procedure and its application to a *C. elegans* model system. The referee suggests using a simple toy model to establish a "ground truth" that would inform our analysis before applying it to the *C. elegans* example.

We view the pairwise maxent model itself as a toy model. It is particularly apt for our calculation because it is an example for which the Fisher information matrix is well understood and straightforward to calculate. Indeed, there is an established "ground truth" given that the FIM

calculation has been done numerous times for the Ising model and other biological and physics models (Oizumi et al., 2011; Transtrum et al. 2010, 2011, 2014, 2015; Machta et al. 2013; Lee et al., 2020; Quinn et al. 2019, 2021; etc.). The calculation of the FIM builds on a large literature of research in theoretical physics, and we would consider this part of the framework to be tested and rigorous.

Then, the most interesting question is how an information geometric framework based on this existing modeling approach can be useful in biological experiments. What remained unexamined before our analysis was (1) how to test a predicted FIM using realizable experimental perturbations, (2) how to interpret the results in terms of two types of sparsity, and (3) how this sparsity is related to the system's interaction structure.

For this last analysis, we consider simpler null models including a homogenous model, an independent model, and shuffled couplings. The homogeneous model as mentioned in the discussion, "Since identical neurons would imply uniform eigenvectors," is discussed in previous work (Lee et al., 2020). We also tested an independent model that show that the interactions are crucial (see Figure 4C for sensitivity measures and Figure E.14 for uniformities). Finally, we considered shuffled versions of the *C. elegans* interaction structure to distinguish the role of the statistics of the interaction structure from the the particular arrangement. These comparisons to simpler versions of our toy model all help refine our claim about what a pivotal neuron is: that it is distinguished by heterogeneity in its bias and its interaction structure and that such heterogeneity comes from the distribution of couplings.

We use *C. elegans* as an example because it is an experimentally accessible neural system whose control structure is still not well understood. Given the number of theoretical *C. elegans* models in the literature that have yet to be related to an experimental "ground truth," we hoped to shortcut the theoretical debate and focus on describing an experiment that could nail observations down. We argue that our main contribution is to make this connection concrete such that we can test its predictions. Indeed, the exercise of specifying the connection to experiment has elucidated some fundamental questions about how to concretely measure and test predictions from the "sloppiness" literature in experiment. Thus, our main goal was to clearly explain the theoretical process while motivating how it could be implemented in a feasible future experiment, which constitute the two parts of the paper.

Have the authors made all data and (if applicable) computational code underlying the findings in their manuscript fully available?
The PLOS Data policy requires authors to make all data and code underlying the findings described in their manuscript fully available without restriction, with rare exception (please refer to the Data Availability Statement in the manuscript PDF file). The data and code should be provided as part of the manuscript or its supporting information, or deposited to a public repository. For example, in addition to summary statistics, the data points behind means, medians and variance measures should be available. If there are restrictions on publicly sharing data or code —e.g. participant privacy or use of data from a third party— those must be specified.

Reviewer #1: No: The dataset had been previously published (although the reference provided in the current manuscript is wrong, see Comments to the Authors), but no explicit link to the data used in this manuscript is provided. Also, the code available on github, but currently there is no section in the main text or appendix pointing to the code. I believe that this information should be stated more explicitly, with direct links to both code and data appearing on the main text (or in Appendix).

We have now included links to our code online. We note that the original analysis cannot be easily run because it requires a supercomputer, and these have customized setups. In "Data & code," we write,

> The code base is available at https://github.com/eltrompetero/maxent_fim and will be archived on Zenodo (link to be added upon finalization of the manuscript). Note that the analysis in the paper requires a supercomputer, which generally require customized scripts. The experimental trials we use are on OSF at https://osf.io/mw5zs/ for reference [64] and at https://osf.io/dpr3h/ for reference [4].
