# Supplementary material for: Discovering sparse control strategies in neural activity
Source: PLoS Comput Biol. 2022 May 27;18(5):e1010072. doi: 10.1371/journal.pcbi.1010072 (PMC9140285; doi:10.1371/journal.pcbi.1010072)
Supplement: Attachment — Submitted filename: Review PCOMPBIOL.pdf [file pcbi.1010072.s004.pdf]

Review PCOMPBIOL-D-21-01485R1
Discovering sparse control strategies in neural activity

The retitled manuscript "Discovering sparse control strategies in neural activity" presents a significantly improved, well-polished exposition of a compelling combination of maximum-entropy modelling and information geometry for finding control strategies in networks of neurons. Leveraging the *C. elegans* brain as a minimal example, this manuscript finds evidence of sparse control mechanisms through experimentally inspired "in-silico" perturbations and an advanced use of information geometry and inference.

The revised manuscript was significantly improved, and I find that the authors have answered most of my concerns in a satisfactory fashion. There are, however, a few points that I'd like to see discussed/addressed before I can fully recommend publication.

1. Regarding point 2a) of my previous review, I would like to add that the measurement time is not the only constraint on the class of behaviors that can be probed by the analysis proposed in this manuscript. Importantly, the choice of discretization and test-statistics also fundamentally constrain the behavioral timescales accessible to this analysis. For example, sequences of stereotyped short-timescale behaviors (such as backward movements or turns) can give rise to different longer time scale manoeuvres (such as pirouettes) which are an important unit of behavior on longer time scales. Structures such as these could in principle be assessed through a different discretization of the neural dynamics, or by considering time expanded sequences of neural states. From the point-of-view of an experimental protocol, given longer neural recordings one would like to understand more complex longer time scale behaviors. Could the authors provide some guidance on how to proceed in this case? This could be useful to strengthen the generality of the approach, but also could provide more practical guidance on the use of these ideas in other contexts.

2. The answer to point 15a) is at least incomplete. As signaled by the authors, the AVA neuron is identified with a blue fluorescent tag in the dataset from (Hallinen et al. eLife 2021), and so one could check if AVA appears as one of the pivotal neurons.

3. The inferred max-ent model is essentially a 3-spin Sherrington-Kirkpatrick (SSK) model with external fields. However, the coupling matrix is likely not from the typical Gaussian Orthogonal Ensemble, and the statistics of the external field are likely non-trivial. Could the authors comments on how the statistics of the couplings and fields found in the *C. elegans* brain compare with classical spin-glass models? What properties of the coupling and fields give rise to the observed information-geometric properties observed in *C. elegans*?

4. In the discussion of Appendix B, it should be stressed that even though a sparse interaction network seems more appropriate, it has been shown that in the C. elegans brain there are crucial extra-synaptic signalling pathways that exert couplings between neurons that are not synaptically connected. For reviews, see e.g. Bentley et al. PLoS Comput Biol 2016 (doi: 10.1371/journal.pcbi.1005283) or Bargmann Bioessays 2012 (doi: 10.1002/bies.201100185), and for a distinct example relevant for short-time behavior see e.g. Lu et al. biorXiv 2022 (doi: 10.1101/2021.09.21.461278).

5. The analysis of Fig. C3 is confusing. How is it possible to obtain negative mutual information in some experiments? Would it perhaps be easier to use a simpler measure of correlation? Also, can the variation in the obtained mutual information measures among worms be compared to the linear model predictions of the mapping between brain and behavior found in Hallinen et al.? This would support the statement that in some experiments the observed neural activity is not related to behavior.

Minor points:

6. In the Discussion section about the limitations of the implementation, the use of the expression dynamical attractors to designate short-timescale behavioral motifs is imprecise. It remains to be shown whether short-time behaviors can be treated as attractors, in a precise dynamical systems theory sense. In fact, detailed analysis of the geometry of state-space trajectories in *C. elegans* has shown that it exhibits signs of deterministic chaos in foraging experiments, suggesting that there is an infinite set of unstable periodic orbits underlying *C. elegans* behavior (Ahamed et al. Nature Physics 2020). I'd suggest that the authors make these sentences more precise, either by not using the term "dynamical attractors" or by expanding the discussion.

7. Comparing Figure B2 and 2, we see that there are significant differences between the two datasets. Could the author's comment on this?

8. In the panel D of Figs2 and B2, what is the reason for not showing the fine-grained synchrony for the shuffled model? That would make the bottom row of figure more uniform.

9. In Fig.3A and its discussion in the text, it is mentioned that optogenetic perturbations are more "elegant", but aren't they also less invasive and easier to use in a set-up where the worms are allowed to move?

10. The highlighted part of the following section is missing a subject: "While synchrony does not differentiate between the orientation of the states, it presents a simple and computationally tractable statistic [77, 78], can contain information about worm posture and velocity (see Appendix C), and is reproduced by the pairwise maxent model as we show in Figure 2D"

11. The following sentence seems to be incomplete: "One major questions that we approach in our framework is how experimental perturbations should be represented in the model."

12. There is no reference to appendix J in the main text.
