# Supplementary material for: Discovering sparse control strategies in neural activity
Source: PLoS Comput Biol. 2022 May 27;18(5):e1010072. doi: 10.1371/journal.pcbi.1010072 (PMC9140285; doi:10.1371/journal.pcbi.1010072)
Supplement: Attachment — Submitted filename: ref response 2.pdf [file pcbi.1010072.s005.pdf]

Dear Editor,

We have gone through the reviewers' additional comments point by point below.

Reviewer 1 raised a number of additional points of scientific interest. While we have addressed most points with appropriate modification of the text, some of the points seemed to be either comments of curiosity or questions that would extend the remit of the paper as it is written. We have done our best to address the comments fully within the context of the work we presented in our original submission.

Sincerely,

Edward D. Lee
Xiaowen Chen
Bryan C. Daniels

Reviewer 1

The retitled manuscript "Discovering sparse control strategies in neural activity" presents a significantly improved, well-polished exposition of a compelling combination of maximum-entropy modelling and information geometry for finding control strategies in networks of neurons. Leveraging the C. elegans brain as a minimal example, this manuscript finds evidence of sparse control mechanisms through experimentally inspired "in-silico" perturbations and an advanced use of information geometry and inference.

The revised manuscript was significantly improved, and I find that the authors have answered most of my concerns in a satisfactory fashion. There are, however, a few points that I'd like to see discussed/addressed before I can fully recommend publication.

We thank the reviewer for their time and attention in providing further constructive feedback, which we address below. We are glad to find that the reviewer considers our response to have answered most of the reviewer's points satisfactorily.

Regarding point 2a) of my previous review, I would like to add that the measurement time is not the only constraint on the class of behaviors that can be probed by the analysis proposed in this manuscript. Importantly, the choice of discretization and test-statistics also fundamentally constrain the behavioral timescales accessible to this analysis. For example, sequences of stereotyped short-timescale behaviors (such as backward movements or turns) can give rise to different longer time scale manoeuvres (such as pirouettes) which are an important unit of behavior on longer time scales. Structures such as these could in principle be assessed through a different discretization of the neural dynamics, or by considering time expanded sequences of neural states. From the point-of-view of an experimental protocol, given longer neural recordings one would like to understand more complex longer time scale behaviors. Could the authors provide some guidance on how to proceed in this case? This could be useful to strengthen the generality of the approach, but also could provide more practical guidance on the use of these ideas in other contexts.

The referee asks how the choice of discretization and test statistics might be altered in a longer experimental timescale to explore more complex behaviors. We think the referee has already started answering the question, "Structures such as these could in principle be assessed through a different discretization of the neural dynamics, or by considering time expanded sequences of neural states."

We note that these choices are currently inputs for our outlined approach. We hope that experimental tests will allow us to eventually check how our results depend on these choices and whether or not some choices are better than others.

We now touch on this question in the discussion,

> Furthermore, we focused on a collective statistic measured at the same short timescales as the neural perturbations, but this could be expanded to measurements over longer timescales coinciding with behaviors of interest.

The answer to point 15a) is at least incomplete. As signaled by the authors, the AVA neuron is identified with a blue fluorescent tag in the dataset from (Hallinen et al. eLife 2021), and so one could check if AVA appears as one of the pivotal neurons.

The AVA neuron is not indicated in the Scholz et al. data set that we use. They only attempt neuron alignment, and as we mentioned in the previous response, one of the authors of that manuscript expressed pessimism about the reliability of the alignment. We rely on this data set because Dr. Chen is well-familiar with its limitations and merits.

We now elaborate on this in Appendix H,

> Given the data set we use, we focus on anterior neural activity, which excludes motor neurons that are directly implicated in actuating muscles and represents about half of the neurons in the mature worm. These neurons are not labeled. Although post-processing may give a rough idea of neural identity, it is of limited reliability.

The inferred max-ent model is essentially a 3-spin Sherrington-Kirkpatrick (SSK) model with external fields. However, the coupling matrix is likely not from the typical Gaussian Orthogonal Ensemble, and the statistics of the external field are likely non-trivial. Could the authors comments on how the statistics of the couplings and fields found in the C. elegans brain compare with classical spin-glass models? What properties of the coupling and fields give rise to the observed information-geometric properties observed in C. elegans?

The inferred model is not the SK model, which would be a mean-field model with all-to-all pairwise couplings i.i.d. sampled from a Gaussian distribution. Our model is sparse and the distribution of couplings is inferred, not given by a Gaussian distribution. Thus, we do not expect classical spin-glass models to describe the system well, and it is unclear whether or not some other theoretical distribution may fare any better.

Furthermore, the relationship between the distribution of couplings and the distribution of entries of the FIM is nontrivial.

Ultimately, we agree that determining statistical properties of the distribution of couplings that lead to the observed information geometry is a logical next step. However, due to the complexity of addressing these questions, we leave them to future work.

In the discussion of Appendix B, it should be stressed that even though a sparse interaction network seems more appropriate, it has been shown that in the C. elegans brain there are crucial extra-synaptic signalling pathways that exert couplings between neurons that are not synaptically connected. For reviews, see e.g. Bentley et al. PLoS Comput Biol 2016 (doi: 10.1371/journal.pcbi.1005283) or Bargmann Bioessays 2012 (doi: 10.1002/bies.201100185), and for a distinct example relevant for short-time behavior see e.g. Lu et al. biorXiv 2022 (doi: 10.1101/2021.09.21.461278).

We have now included this information as a paragraph in Appendix B:

> We note that signalling pathways have been found between neurons in the *C. elegans* brain that are not synaptically connected. For reviews, see references [122] and [123], and for a distinct example relevant for short-time behavior see reference [124].

The analysis of Fig. C3 is confusing. How is it possible to obtain negative mutual information in some experiments? Would it perhaps be easier to use a simpler measure of correlation? Also, can the variation in the obtained mutual information measures among worms be compared to the linear model predictions of the mapping between brain and behavior found in Hallinen et al.? This would support the statement that in some experiments the observed neural activity is not related to behavior.

Negative estimated values for the mutual information arise from an issue of precision. Since the mutual information between two random variables X and Y can be expressed as H[X] + H[Y] - H[X,Y], and we estimate each term independently of the others, mutual informations near zero can sometimes be estimated as negative values. This is a general problem for estimation of mutual information, but we would hope that a good estimator would be consistent with non-negative values within error bars. This is the case with a single exception in experiment 162140, which provides a check that our approach is reasonable. We now mention this in the caption of Figure C.3,

> Error bars for synchrony MI are given by the NSB estimator for the joint and independent entropies separately [117]. This means our estimate for MI can be negative but is reassuringly consistent with non-negative values within error bars in every case except one.

The advantage of using an information-theoretic quantity is that it captures nonlinear dependence, whereas a measure of correlation would not.

While the referee's suggestion to compare our mutual information estimate with the linear model predictions in Hallinen et al. might be an interesting point of contrast, these present two different measures. Importantly, our calculation does not mean that "observed neural activity is not related to behavior," but that the particular coarse-graining that we chose is not related to behavior. This is not inconsistent with the fact that Hallinen et al. found behavioral predictors. Such a comparison is not directly relevant to the work that we present here.

Minor points:

In the Discussion section about the limitations of the implementation, the use of the expression dynamical attractors to designate short-timescale behavioral motifs is imprecise. It remains to be shown whether short-time behaviors can be treated as attractors, in a precise dynamical systems theory sense. In fact, detailed analysis of the geometry of state-space trajectories in C. elegans has shown that it exhibits signs of deterministic chaos in foraging experiments, suggesting that there is an infinite set of unstable periodic orbits underlying C. elegans behavior (Ahamed et al. Nature Physics 2020). I'd suggest that the authors make these sentences more precise, either by not using the term "dynamical attractors" or by expanding the discussion.

We have modified the text,

> The experiments we consider last on the order of 10^2 s, and so pairwise correlations can reflect neural activity on the timescale of several correlated behaviors including much faster head casts, bending, and even multiple locomotory reversals and the transitions between them [65,90]. In this sense, perturbations indicate modifications to neural synchrony representative of this mixture and likely not of any single stereotyped behavior.

Comparing Figure B2 and 2, we see that there are significant differences between the two datasets. Could the author's comment on this?

It is likely that the two experiments are measuring different neurons. That different ensembles from the same region show similar patterns ofsensitivity is what we aim to show with this comparison.

In the panel D of Figs2 and B2, what is the reason for not showing the fine-grained synchrony for the shuffled model? That would make the bottom row of figure more uniform.

We do not show the shuffled models to keep the figure uncluttered. The point of panel D is to indicate that the pairwise model matches fine-grained synchrony well and much better than the independent model, which the current version does.

In the course of answering this point, we realized that we had repeated the fit to the alternative experiment featured in the appendices and have fixed this problem.

In Fig.3A and its discussion in the text, it is mentioned that optogenetic perturbations are more "elegant", but aren't they also less invasive and easier to use in a set-up where the worms are allowed to move?

We have modified the text to be more specific about what we meant by "elegant,"

> In a hypothetical experiment, we might effect perturbations by inserting electrodes into immobilized worms or, in a *less invasive and more natural protocol,* by using optogenetic tools…

The highlighted part of the following section is missing a subject: "While synchrony does not differentiate between the orientation of the states, it presents a simple and computationally tractable statistic [77, 78], can contain information about worm posture and velocity (see Appendix C), and is reproduced by the pairwise maxent model as we show in Figure 2D"

The subject in this sentence is "synchrony" as indicated by the pronoun "it."

The following sentence seems to be incomplete: "One major questions that we approach in our framework is how experimental perturbations should be represented in the model."

This typo has been corrected: "One major question that we approach in our framework is how experimental perturbations should be represented in the model."

There is no reference to appendix J in the main text.

We do not find it necessary to refer to this appendix in the main text.

Reviewer #2

The authors have addressed my comments.

I have a few remaining textual changes to improves the accuracy of the terms used.

We thank the reviewer for their detailed feedback, which we have incorporated as described below.

"making small perturbations to internal states at the level of individual components"
Internal states has a distinct meaning in neuroscience, e.g, motivation, hunger, sleepiness ... which could be confusing to the reader in this sentence. The authors should just use neural activity in its place here.

We have adjusted the text accordingly:

> Experimentally, our method requires measuring and making small perturbations to *neural activity* at the level of individual components, along with the simultaneous measurement of a collective state or output behavior.

Later in the same paragraph, the authors even refer to that meaning of 'internal state', but also use gene expression in the list. I would recommend staying with 'microscopic quantities' or similar to avoid any confusion.

We have removed the later reference to "internal states" as well:

> One could imagine adapting the method to hormone concentration, gene expression, or other experimentally accessible quantities [92–97] and to output behavior including morphological descriptors [42,98,99], a stereotyped behavioral sequence [51,100,101], or developmental outcomes.

"the anterior neural network" - this should be anatomically correctly named as there is no such thing as an anterior neural network in C. elegans: a subset neurons from the anterior ganglia in the worm head or
simpler 'neurons located in the anterior of the worm'

We have modified the text to

> The two examples we consider are of N = 50 neuron subsets from the neurons located in the anterior of the immobilized worm from reference [65].

Minor: References to "Theoretical formulation." should be replaced with the new section title

This has been done.
